# The sensitivity of normal brain and intracranially implanted VX2 tumour to interstitial photodynamic therapy.

**DOI:** 10.1038/bjc.1996.58

**Published:** 1996-02

**Authors:** L. Lilge, M. C. Olivo, S. W. Schatz, J. A. MaGuire, M. S. Patterson, B. C. Wilson

**Affiliations:** McMaster University, Hamilton ONT, Canada.

## Abstract

**Images:**


British Journal of Cancer (1996) 73, 332-343

© 1996 Stockton Press All rights reserved 0007-0920/96 $12.00

### The sensitivity of normal brain and intracranially implanted VX2 tumour to interstitial photodynamic therapy

L Lilge[1,2], MC Olivo[1,2], SW Schatz[1,3], JA MaGuire[1,4], MS Patterson[1,2] and BC Wilson[5]

[1]*McMaster University, 1280 Main Street West, Hamilton ONT L8S 4M8, Canada;* [2]*Hamilton Regional Cancer Centre, 699 Concession Street, Hamilton ONT L8V 5C2; Hamilton General Hospital, Canada;* [3]*Service of Neurosurgery and* [4]*Service of Neuropathology, 237 Barton Street E, Hamilton ONT L8L 2X2, Canada;* [5]*Ontario Cancer Institute/Princess Margaret Hospital and Department of Medical Biophysics, University of Toronto, 6120 University Avenue, Toronto, Ontario M5G 2M9, Canada.*

**Summary** The applicability and limitations of a photodynamic threshold model, used to describe quantitatively the *in vivo* response of tissues to photodynamic therapy, are currently being investigated in a variety of normal and malignant tumour tissues. The model states that tissue necrosis occurs when the number of photons absorbed by the photosensitiser per unit tissue volume exceeds a threshold. New Zealand White rabbits were sensitised with porphyrin-based photosensitisers. Normal brain or intracranially implanted VX2 tumours were illuminated via an optical fibre placed into the tissue at craniotomy. The light fluence distribution in the tissue was measured by multiple interstitial optical fibre detectors. The tissue concentration of the photosensitiser was determined post mortem by absorption spectroscopy. The derived photodynamic threshold values for normal brain are significantly lower than for VX2 tumour for all photosensitisers examined. Neuronal damage is evident beyond the zone of frank necrosis. For Photofrin the threshold decreases with time delay between photosensitiser administration and light treatment. No significant difference in threshold is found between Photofrin and haematoporphyrin derivative. The threshold in normal brain (grey matter) is lowest for sensitisation by 5δ-aminolaevulinic acid. The results confirm the very high sensitivity of normal brain to porphyrin photodynamic therapy and show the importance of *in situ* light fluence monitoring during photodynamic irradiation.

**Keywords:** photodynamic therapy; porphyrin; threshold model

Photodynamic therapy (PDT), a treatment using a light-activated drug (photosensitiser), is an investigational therapy for several forms of cancer (Marcus and Dugan, 1992). The photosensitiser, selectively accumulated or retained in neoplastic tissue, produces cytotoxic substances upon activation with light, resulting typically in gross haemorrhagic tissue necrosis.

Intraoperative local treatment of primary or recurrent malignant brain tumours by PDT is currently being investigated, and several phase I/II clinical trials have been reported with evidence of increased survival time (Kostron *et al.*, 1990; Muller and Wilson, 1991; Kaye and Hill, 1993). Depending on the clinical situation, tumour mass, location and previous local treatments, PDT is given with or without prior surgical resection of the tumour and either as a stand-alone treatment (Muller and Wilson, 1990; Powers *et al.*, 1991) or in combination with radiation or chemotherapy (Kaye, 1989; Noske *et al.*, 1991). Both intracavitary (Muller and Wilson, 1993) and interstitial techniques (Muller and Wilson, 1991; Powers *et al.*, 1991) have been used. A critical issue with either technique is the degree of tumour selectivity that can be achieved. The uptake of photosensitiser into tumour may be consistently greater than in normal brain, owing to the intact blood-brain barrier (BBB) of the latter. However, low intrinsic sensitivity of normal brain compared with malignant tissue is also desirable in order to minimise damage to brain adjacent to tumour (BAT), which is within the light treatment field. Haemorrhagic necrosis of malignant and normal brain tissue in patients following PDT has been reported (Muller and Wilson, 1990; Kaye and Hill, 1993).

In this preclinical study a photodynamic threshold model has been used to describe the dependence of the extent of PDT-induced tissue necrosis on light and photosensitiser doses. The model (Patterson *et al.*, 1990) was derived from the observation of a sharp boundary that usually occurs between PDT-induced tissue necrosis and undamaged adjacent tissue (Berenbaum *et al.*, 1982; Potter, 1989). The model states that, for a given tissue and photosensitiser combination, necrosis will occur if the number of photons absorbed by the photosensitiser per unit tissue volume exceeds a threshold value ($T$). Thus, a low intrinsic PDT sensitivity corresponds to a high $T$-value, and vice versa. The photodynamic threshold is calculated from measured values of the depth (or radius) of coagulative tissue necrosis ($r_c$), the light fluence [$H(r_c)$] at the necrosis boundary, and the photosensitiser concentration in the tissue ($C$). The model has been tested in various *in vivo* animal tumour models and normal tissues (Farrell *et al.*, 1991, 1992*a*; Q Chen, private communication) and has been shown to predict accurately the radius or depth of necrosis.

Only a limited number of quantitative studies describing intracranial tissue response to PDT in preclinical animal models have been published. Evidence to date (Dereski *et al.*, 1991; Chen *et al.*, 1992*a,b*; Lilge *et al.*, 1993*a*; Ying *et al.*, 1994) suggests that the photodynamic threshold model holds also for intracranial tissues. Kaye and Morstyn (1987), using haematoporphyrin derivative (HpD) at 20 mg per kg body weight (bw) and 200 J cm$^{-2}$ of surface light dose, and Sandeman *et al.* (1987), using trisulphonated aluminium chlorophthalocyanine at 0.5 mg per kg bw and 700 J cm$^{-2}$, observed selective tumour kill in intracranial murine tumour models, while normal brain tissue showed no, or very limited necrosis under these treatment conditions.

Several authors (Cheng *et al.*, 1984; Dereski *et al.*, 1991; Chen *et al.*, 1992*a*; Leach *et al.*, 1993; Ying *et al.*, 1994) have noted a very high sensitivity of normal brain tissue to PDT using Photofrin as photosensitiser. The first histologically observable changes in normal brain are in structures associated with the BBB, such as endothelial cell damage (Berenbaum *et al.*, 1986) and astrocytic swelling (Yoshida *et al.*, 1992*a*). Neuronal injury (Yoshida *et al.*, 1992*b*) was observed only at longer time delays post PDT. Lindsay *et al.* (1991) observed photodynamic resistance for intra-

Correspondence: BC Wilson, Department of Medical Physics, Ontario Cancer Institute/Princess Margaret Hospital and Department of Medical Biophysics, University of Toronto, 6120 University Avenue, Toronto, Ontario M5G 2M9, Canada
Received 15 March 1995; revised 18 July 1995; accepted 25 July 1995

cranially grown VMDk tumours in mice, while the same tumour grown subcutaneously appeared sensitive using tetra-*m*-(hydroxyphenyl) porphyrin (*m*-THPP) as photosensitiser.

These various data suggest differences in the threshold values of porphyrin-based photosensitisers in the photodynamic response of normal brain tissue, and in particular a higher threshold for HpD compared with Photofrin. [Photofrin (Quadralogic Technologies, Vancouver, BC, Canada) or porfimer sodium, is a commercial version of HpD that is enriched in components believed to have enhanced tumour localisation and high PDT activity.]

The aim of the present study was to determine and compare the PDT thresholds for intracranial PDT of normal brain and an implanted tumour model in the rabbit brain for three porphyrin-based photosensitisers, namely Photofrin, HpD and 5$\delta$-aminolaevulinic acid (5-ALA), a precursor of the haem synthesis pathway that leads to the *in situ* production of the photosensitiser protoporphyrin IX (PpIX) (Kennedy and Pottier, 1992). Additionally, the feasibility of using an optical fibre detector array for interstitial fluence-rate monitoring and determination of tissue optical properties *in vivo* is demonstrated.

### Materials and methods

### *Animal model and preparation*

Experiments were performed in male New Zealand White (NZW) rabbits, 2.5–3.8 kg bw. The animals were randomised for PDT treatment according to (i) implanted VX2 tumour or normal brain; (ii) photosensitiser type; and (iii) time delay between photosensitiser injection and light irradiation. Table I lists the five study groups. Three animals received light treatment without prior photosensitiser injection in order to investigate possible tissue effects from heating caused by light absorption or from photoactivation of endogenous porphyrins. In a further rabbit interstitial temperature measurements in normal brain were made to check the possibility of temperature changes during light irradiation.

The VX2 tumour is a high-grade carcinoma derived from a virus-induced papilloma in a domestic rabbit in 1937 (Carson *et al.*, 1982). For these experiments the cell line was propagated i.m. in the flank of NZW rabbits with monthly passages. The experiments extended over a 15 month period.

For tumour implant into the brain, a cell suspension was prepared from freshly excised VX2 tumour in sterile phosphate-buffered saline (PBS). The number of VX2 cells (approximately $10^5$) required to grow an intracerebral tumour of approximately 10–14 mm diameter in 14 days was determined in preliminary experiments. For tumour induction the animals were anaesthetised with i.m. injection of 1 mg per kg bw acepromazine (Atravet; Ayerot, Montreal, Quebec, Canada) followed by 5 mg per kg bw xylazine (Rompun; Haver-Lockhart, Etobicoke, Ontario, Canada), and 50 mg per kg bw ketamine (Ketalean; MTC Pharmaceuticals, London, Ontario, Canada). For local anaesthesia 1 ml of 2% xylocaine was injected into the scalp. The skull was exposed and a 3 mm burr hole drilled in the left parietal bone, 5 mm posterior to the coronal suture and 5 mm lateral to the sagittal suture. A 25–50 $\mu$l cell suspension containing approximately $10^5$ cells was injected through the burr hole into the brain to a depth of 5–6 mm using a 25 G needle (Brem *et al.*, 1990). After injection the burr hole was closed with bonewax and the scalp incision sutured. After 14 days, before PDT, on inspection of the brain cortex at craniotomy, tumour was sometimes visible at the injection site and in some cases also attached to the dura. This 'leakage' of tumour cells, which occurred mainly in the first few animals in which the injection technique had not been perfected, did not cause any obvious inflammatory response and the bulk of the tumour mass remained well defined. The problem could be eliminated by implantation of the cells in agarose gel as reported by Kaye *et al.* (1985).

### *PDT treatment*

Photofrin was injected i.v., 10 mg per kg bw at 4–6, 24 or 48 h before light activation. For comparison, two animals were injected i.v. with HpD (supplied by Queen Elizabeth Hospital, Woodville, Australia) at 10 mg per kg bw 24 h before light activation, and three animals were injected i.v. with 5-ALA as hydrochloride (98% purity) (Sigma Scientific, St Louis, MO, USA) at 100 mg per kg bw 6 h before light activation. These injected doses are in the range of commonly used doses in preclinical research (Dereski *et al.*, 1991; Chen *et al.*, 1992*a*; Yoshida *et al.*, 1992*a*). For i.v. injection, Photofrin and HpD solutions were made up in 5% dextrose to 10 mg ml$^{-1}$ and 5 mg ml$^{-1}$ respectively. 5-ALA was made up in PBS to 80 mg ml$^{-1}$ and pH balanced to 7.1 with sodium hydroxide (0.1 N).

An air cooled argon–ion laser (Ion Laser Technologies, Salt Lake City, UT, USA) operating in multiline mode ($\lambda$=465/488/514 nm) or single-line mode ($\lambda$=514 nm) was used as the light source [630 nm is commonly used for these photosensitisers, but a shorter wavelength was chosen in order to confine the treatment to within the tumour volume; previous work has shown that this does not change the measured threshold values (Patterson *et al.*, 1990)]. The light was coupled via a microscope objective lens into a fused silica optical fibre (numerical aperture 0.25; 320 $\mu$m core diameter, 415 $\mu$m outer diameter; Polymicro Technologies, Phoenix, AZ, USA). The numerical aperture was slightly overfilled at the input face to obtain a reproducible angular distribution of the light at the cleaved and polished distal end. For introduction into the cranium the fibre was held flush with the end of a 21 G biopsy needle. The delivered power was measured with a thermopile (Scientech, Boulder, CO, USA) immediately before and after irradiation, in all cases varying less than 6% between these two measurements after cleaning the tip. All source and detector fibres were sterilised for 15 min in 0.85% sodium hydroxide solution and rinsed in sterile saline immediately before use.

The light fluence (J cm$^{-2}$) at the anticipated boundary of necrosis was determined by integrating direct continuous measurements of the local fluence-rate in the target volume during PDT (Powers and Brown, 1986; Lilge and Wilson, 1993) using three implanted optical fibre probes. These fluorescent (rhodamine 640) tipped probes (170 $\mu$m outer

**Table I** Summary of study groups

| Group | Photosensitiser | Intravenous photosensitiser dose mg kg$^{-1}$ | No. of animals Tumour-bearing | Normal | Treatment time interval (h) | Delivered light energy (J) |
|---|---|---|---|---|---|---|
| 1 | Photofrin | 10 | 2 | 2 | 4-6 | 77–100 |
| 2 | Photofrin | 10 | 6 | 4 | 24 | 100 |
| 3 | Photofrin | 10 | 3 | 2 | 48 | 82–100 |
| 4 | HpD | 10 | 0 | 2 | 24 | 90–100 |
| 5 | 5-ALA | 100 | 1 | 2 | 6 | 100 |
| Control | None | – | – | 3 | – | 100 |

diameter) have been described elsewhere and were calibrated for absolute fluence-rate response (Lilge *et al.*, 1993*b*). They were mounted in 26 G needles in a template along a line parallel to and at the same depth as the treatment fibre, at radial distances, $r$, of 2, 4 and 6 mm (Figure 1). This allowed easy alignment to avoid severing of superficial blood vessels of the cortex. The probe outputs were imaged onto a CCD array (Photometrics, Tuscon, AZ, USA) for simultaneous collection from the three positions in the tissue. A semilogarithmic plot of the product of fluence times radial distance *vs* radial distance from the source showed a linear relationship, the negative slope of which yields the effective attenuation coefficient, $\mu_{eff}$, of the tissue at the treatment wavelength. [This relationship comes from the fact that the fluence rate decreases with distance from the source according to the combination of exponential attenuation and a $1/r$ geometrical factor (Driver *et al.*, 1991).] From this plot, the fluence [$H(r_c)$] at the radius of necrosis, $r_c$, in each animal was determined by interpolation.

Surgical preparation and light treatment were performed under aseptic conditions. For light treatment the animals were anaesthetised as described above. During surgery inhalation of isoflurane (Forane; Anaquest, Mississauga, Ontaria, Canada) at 2–3.5% (v/v) in either oxygen (100%) or in an oxygen–nitrous oxide mixture (30–70%) was used as required to maintain anaesthesia. The gas flow rate was adjusted to 2.5 $min^{-1}$. The oxygen saturation and partial pressure in arterial blood ($paO_2$) were measured in selected rabbits breathing 100% oxygen before and after intracranial light treatment. The oxygen saturation was found to be 99.8% at $paO_2$ of 43.1–51.4 kPa before and after surgery (Albritton, 1953). Surgery for intracranial light irradiation required approximately 1.2–2 h. The skull was exposed and a left parietal craniotomy of 12–15 mm diameter performed. The dura was incised and reflected. The source and detector fibres were positioned under micrometer control to a depth of 6 mm below the cortex surface via biopsy needles. The needles were then retracted, leaving the optical fibres embedded in the white matter, 3–5 mm from the left hippocampus. In tumour-bearing animals the delivery fibre was placed as close as possible to the initial tumour cell injection site and depth, attempting to target the centre of the tumour. The detector fibres were placed posteriorly or posteromedially to the delivery fibre.

After irradiation the optical fibres were retracted and the scalp was sutured. Recovery from anaesthesia was continuously monitored. Dexamethasone 0.5 mg twice daily and enrofloxacin (Baytril; Haver-Lockhart, Etobicoke, Ontario, Canada) as antibiotic 12.5 mg daily were administered i.m.

The surface of the brain was exposed to a maximum broad-band irradiance of 3.6 J $cm^{-2}$ (approximately 7 J total energy) from ambient operating room light. At 6 mm depth the contribution of this to the treatment fluence was negligible (<1%) and could not be detected by the implanted detector probes.

The first five animals in the treatment groups were irradiated with approximately 55 mW delivered power (multiline mode) to minimise the exposure time. However, occasional charring at the delivery fibre tip was observed, and the output was reduced to 42–48 mW (single line) for all subsequent treatments, with no further charring or damage to the fibres. Delivery of 77–100 J total energy took up to 35 min.

Even although this extreme temperature effect at the fibre tip had been eliminated, there remained the possibility that the PDT treatment light could induce hyperthermia. Normal brain tissue is highly temperature sensitive, and hyperthermia approximately 40–42°C has been shown to be synergistic with PDT (Dereski *et al.*, 1995*a*) and can lower PDT threshold values (Farrell *et al.*, 1991). Hence, in an additional non-tumour-bearing rabbit *in situ* temperature measurements were made in the brain by inserting three calibrated type-T thermopiles via 25 G needles at 2, 4 and 6 mm from the source fibre at the same depth as the detector fibre array in the treatment groups. All other procedures were as for the PDT treatments, except that no photosensitiser was given and no detector fibres were used. Five minutes after inserting the temperature probes, measurements were made for periods of 15 min at each of 35, 46, 52, 60 and 80 mW delivered power. At 80 mW the maximum temperature increase, at the 2 mm position, was 1.5°C. At 60 mW and below, the maximum rise recorded

**Figure 1** Experimental set-up for intracranial PDT treatments and light fluence measurements. The delivery and detector fibres were inserted under micrometer control to a depth of 6 mm below the brain surface, into either the white matter or the tumour mass.

was less than 1°C at any time or position. Thus, hyperthermia should not have been a significant factor in the tissue response under the irradiation conditions used.

*Determination of the radius of PDT-induced tissue necrosis*

The animals were sacrificed 24–48 h post PDT treatment (Yoshida *et al.*, 1992*b*) by i.v. sodium pentobarbital overdose (Euthanyl; Haver-Lockhart, Etobicoke, Ontario, Canada). The brain was immediately removed intact and placed in 10% formalin in PBS. After fixation the brain was sliced into transverse sections 3 mm thick for gross examination. Serial sections (4 μm) were cut from those transverse slices that showed the maximum area of necrosis. These were stained with haematoxylin and eosin (H&E) to enhance coagulative necrosis.

The area of induced necrosis, in a 2-D section, was determined by video microscopy at 25× magnification using a colour CCD camera (Javelin, Japan) and a computer-based image analysis program (Bioquant; R&M Biometrics, Nashville, TN, USA). Necrosis was often delineated by the lateral ventricle or a sulcus, causing non-spherical lesions. The 'effective' radius of necrosis was calculated assuming a spherical lesion with a maximum cross-sectional area equal to the measured area.

In tumour-bearing animals, some necrosis of normal brain from PDT was inevitable, and the area of necrosis comprised both normal and tumour tissue. It has been reported (Dereski *et al.*, 1991; Yoshida *et al.*, 1992*b*) that the area of neuronal damage in normal brain usually extends beyond the area of frank coagulative necrosis. This was, therefore, also scored in each animal. The area of frank necrosis corresponds to 'intended' damage in tumour, while neuronal damage is the concomitant extent of damage in adjacent normal brain.

*Determination of photosensitiser concentration in tissue*

For photosensitiser uptake studies one tumour-bearing animal was sacrificed by i.v. euthanyl overdose at each light treatment time point after photosensitiser injection. The brain was immediately removed and stored at −80°C until further use. The right hemisphere was regarded as normal brain (N) and the mid-section of the left hemisphere as tumour (T). The anterior and posterior parts of the left hemisphere were treated as brain adjacent to tumour (BAT). Aliquots of 100–200 mg of each tissue were weighed and the porphyrins extracted with ether (Mang *et al.*, 1987). The porphyrin concentration was then determined by optical absorption spectroscopy at the Soret band (403 nm), to measure total porphyrin content in the tissue, calibrated by spiking a corresponding control homogenised tissue sample with a known concentration of either Photofrin, HpD or PpIX free acid, each dissolved in a 1:1 mixture of 0.01 N tetrahydrofuran and 0.1 N hydrochloric acid. Since this chemical extraction does not differentiate between exogenous and endogenous porphyrins, the concentration of the latter was measured in tissues from an unsensitised rabbit and subtracted from the uptake in the photosensitiser-injected animals. The use of absorption spectroscopy for photosensitiser quantification rather than fluorescence spectroscopy (Vernon *et al.*, 1995) allows the total porphyrin content to be determined, independent of differences in fluorescence yield of the different porphyrin moieties in Photofrin and HpD. It has the disadvantage of lower sensitivity than the fluorimetric technique. The assumption that endogenous porphyrins do not contribute significantly to the photodynamic effect is supported by observations in the light-only control animals (see below).

*Determination of the photodynamic threshold value*

The photodynamic threshold value, T (photons $cm^{-3}$), was calculated (Patterson *et al.*, 1990) according to

$$T = 2.3\epsilon CH(r_c) \qquad (1)$$

where $\varepsilon$ is the extinction coefficient ($cm^{-1}$ ($\mu g\ g^{-1})^{-1}$) of the photosensitiser at the treatment wavelength, C ($\mu g\ g^{-1}$) is the tissue concentration of the photosensitiser and $H(r_c)$ (photons $cm^{-2}$) is the photon fluence at the radius of necrosis $r_c$ (mm). By defining the photodynamic threshold in units of photons $cm^{-3}$ it becomes independent of the treatment wavelength. The extinction coefficients of the photosensitisers were measured in dilute solutions (of dextran for Photofrin and HpD, PBS for ALA) in a double-beam spectrophotometer in the green-to-red part of the visible spectrum. The measured values at the treatment wavelength of 514 nm compare well with those in other studies (Profio and Doiron, 1981; Gunter *et al.*, 1989): Photofrin = 0.011, HpD = 0.038 and PpIX = 0.0002 $cm^{-1}$ ($\mu g\ g^{-1})^{-1}$. The photosensitiser concentrations in tissue were determined as average values in each study group. A quantum energy of 2.45 eV was used to convert J $cm^{-3}$ into photons $cm^{-3}$ for the threshold calculation.

The photodynamic threshold was calculated for each animal based on the individually measured radius of tissue damage, the fluence at this radius and the group-average values for the photosensitiser concentration. Values for $r_c$ and $T$ are presented as mean ± 1 SD within each study group.

### Results

A total of 27 evaluable rabbits received intracranial PDT and local fluence-rate measurements. One animal died of unknown cause within 12 h of PDT treatment. In the three control animals (light–no photosensitiser) only mechanical damage from insertion of the fibre array was observed, with no regional necrosis or zone of neuronal damage seen on histopathology.

As seen in Table II, the tumour-to-normal brain ratio for Photofrin uptake was approximately 12:1 between 4 and 48 h. Highest porphyrin concentrations were found at short time delays, probably due to circulating drug. The data also suggest that uptake of HpD in normal brain is approximately the same as for Photofrin, consistent with previous findings (Wilson *et al.*, 1988).

Different parts of the brain are involved in haem synthesis (Verma *et al.*, 1993), converting 5-ALA to PpIX. Regional differences in synthesis of PpIX from 5-ALA in normal brain have been observed (M Olivo, unpublished results). However,

**Table II** Photosensitiser uptake for the different study groups after subtracting the control values in each tissue; each data point represents a single tissue sample

| Group | Photosensitiser | *Injected dose ($mg\ kg^{-1}$)* | *Time interval (h)* | *Tumour ($\mu g\ g^{-1}$)* | *Brain ($\mu g\ g^{-1}$)* | *BAT ($\mu g\ g^{-1}$)* |
|---|---|---|---|---|---|---|
| 1 | Photofrin | 10 | 4-6 | 13.0 | 1.0 | 1.2 |
| 2 | Photofrin | 10 | 24 | 8.1 | 0.7 | 2.2 |
| 3 | Photofrin | 10 | 48 | 7.0 | 0.5 | 2.1 |
| 4 | HpD | 10 | 24 | NA | 0.5 | NA |
| 5 | 5-ALA | 100 | 6 | 10.3[a] | 1.5[a] | 1.7[a] |
| Control | None | 0 | – | 0.1 | <0.1[b] | 0.1 |

[a] Concentration of PpIX.
[b] Lower detection limit 0.1 $\mu g\ g^{-1}$.
NA, not available.

the bulk sampling of tissue done here does not distinguish specific areas involved in PpIX synthesis. Thus, the data in Table II for PpIX are average tissue values. The PpIX tumour-to-normal brain ratio was approximately 6 : 1 at 6 h after 5-ALA injection.

Figure 2 shows examples of the measured fluence rates, $\Phi(r)$, as a function of time during light treatment. The measured fluence rates changed by up to 50% during the course of treatment in individual animals, but no systematic pattern was observed in any of the study groups. As discussed in the appendix, variations in the local fluence rate, as for example in Figure 2c at 300 s, are most likely due to changes in the local optical properties, rather than fluctuations in delivered power, which would cause a corresponding change in all detector readings. Based on the measured fluence rates, $\Phi(r)$, the total integrated fluence, $H(r)$, at each radial distance, $H(r)$ from the source fibre was calculated and plotted as $r\ H(r)$ *vs* $r$ for each animal, as illustrated in Figure 3. The fluence (J $cm^{-2}$) at the damage radius could thereby be determined in each animal by linear interpolation of these data, and was typically in the range 10–14 J $cm^{-2}$. The fluence rate *vs* distance measurements also allowed the optical absorption and scattering coefficients of the tissues to be determined, as discussed in the appendix.

Figure 4a shows a gross section of VX2 tumour. Histology in tumour-bearing animals showed typical high-grade

**Figure 2** Local fluence rate at 2 mm (■), 4 mm (○) and 6 mm (△) from the source fibre as a function of illumination time during PDT treatment in three separate animals. (**a**) Normal brain, Photofrin at 48 h. (**b**) Tumour, no photosensitiser. (**c**) Tumour, Photofrin at 4 h.

**Figure 3** Example of a graph of the product (fluence × radius) *vs* radius, measured by interstitial fluence-rate probes positioned at radii of 2, 4 and 6 mm from the source fibre tip. The fluences were calculated from the measured fluence rates integrated over the treatment time. The data points are for the same animals as in Figure 2. ■, Normal brain Photofrin at 48 h; ●, tumour, no photosensitiser; ▲, tumour, Photofrin at 4 h. The lines are linear regression fits, the slopes of which yield the effective attenuation coefficients, $\mu_{eff}$, for each tissue. The white arrows indicate the histologically determined radii of coagulative necrosis in the tumour and normal brain of PDT-treated animals. The black arrow indicates the radius of neuronal damage in normal brain.

**Figure 4** Gross histology of VX2 tumour, showing (**a**) the invasion into normal brain pretreatment and (**b**) the extent of coagulative necrosis 24 h after PDT with Photofrin at 24 h.

carcinoma, growing along the major vessels, filling the perivascular space and within the leptomeninges, and with an oedematous BAT region. Figure 4b shows a gross histological section of normal rabbit brain 24 h post PDT-treatment. The PDT-induced lesions in normal brain were characterised by areas of coagulative necrosis with a distinct boundary (Figure 5a). This area of necrosis was bordered by a rim of vacuoles, interpreted as a border of cerebral oedema. This was followed by a thin rim of non-viable neurons, which appeared shrunken and eosinophilic (Figure 6). The thickness of this rim was generally 200–500 $\mu$m, independent of the photosensitiser type or time delay between its injection and light irradiation.

**Figure 5** H&E-stained micrographs of (**a**) normal brain 24 h after PDT with Photofrin at 24 h, (**b**) VX2 tumour before treatment and (**c**) VX2 tumour 24 h after PDT with Photofrin at 24 h. Necrosis in normal brain is surrounded by a rim of vacuoles representing a border of cerebral oedema (**a**; large arrows) and blood vessels showing fibrinoid necrosis (**a**; small arrows). The VX2 tumour is seen growing along the vasculature (**b**; arrow). Nests of viable tumour after PDT are visible within the necrotic volume (**c**; arrows).

In the tumour-bearing animals, nests and cords of viable tumour were observed occasionally within brain tissue adjacent to the PDT-induced necrotic lesions (Figure 5c). Blood vessels within the lesions displayed fibrinoid necrosis in both tumour and normal tissue. Occasional vessels displayed features consistent with vasculitis: polymorphonuclear leucocytes and mononuclear cells infiltrated throughout the full thickness of vessel walls, and several vessels were occluded by fibrin. Choroid plexus within and immediately adjacent to the lesions frequently showed subepithelial accumulations of fibrin and proteinaceous material. No significant differences in the vascular effects were observed for the different time delays between photosensitiser injection and treatment.

No quantitative differences were found in either gross or stained sections between HpD- and Photofrin-injected animals. With 5-ALA, necrosis of normal brain could be found only in the grey matter (Figure 7a), appearing similar to Photofrin-induced coagulative necrosis in the grey matter. No necrosis or subnecrotic effects were found in the white matter.

The measured radii of tissue necrosis, $r_c$, in the different study groups are given in Table III. There was little dependence of $r_c$ with time interval. No statistically significant difference was observed between Photofrin and HpD treated at 24 h. No statistically significant difference in tissue response of animals receiving 100% oxygen or the oxygen/nitrous oxide mixture was observed for normal or tumour-bearing animals (data not shown).

As seen in Table III there was some inter-animal variability in the radius of necrosis. Vascular damage and shutdown contributes to PDT-induced coagulative necrosis and can account for non-spherical lesions and significant intra-animal variation in the extent of coagulative damage. Patchy uptake of the photosensitiser (Boggan *et al*., 1984; Hebeda *et al*., 1995), not observable in our volume-average uptake measurements, could also contribute to non-spherical lesions and irregular appearance of tissue necrosis.

Based on the total fluence at the radius of necrosis, the concentration of the photosensitiser in the tissue and its molar extinction coefficient, the photodynamic threshold value was calculated for each animal according to equation (1). The average values for each study group are listed in Table IV. The photodynamic thresholds for Photofrin- and HpD-treated animals at 24 h were similar.

As a result of the absence of PDT-damage in the white matter of ALA-treated animals, the zone of necrosis was not spherical with the fibre tip at the centre. The upper limit to the threshold value given in Table IV was calculated using a value for the radius of necrosis derived from $r_c^2 = A/4\,\pi$ where $A$ is the maximum cross sectional area of necrosis. The lower

**Figure 6** H&E-stained micrograph of PDT-induced necrosis in normal brain 24 h after treatment with Photofrin at 24 h. Damage to the pyramidal neurons in the hippocampal region (large arrow) can be observed beyond the boundary of coagulative necrosis (small arrow).

**Figure 7** H&E-stained micrographs of PDT-induced necrosis in normal brain 24 h after treatment with 5-ALA at 6 h. (**a**) Necrosis in a grey matter region 5–6 mm from the fibre tip. (**b**) White matter shows only necrosis related to mechanical damage caused by fibre insertion (arrow).

**Table III** Radius of necrosis in each study group: group average ± standard deviation

| Group | Photosensitiser | Treatment time (h) | $r_c$ (mm) VX2 tumour | Normal brain |
|---|---|---|---|---|
| 1 | Photofrin | 4-6 | $4.3\pm0.6$ | $4.3\pm0.7$ |
| 2 | Photofrin | 24 | $3.6\pm0.5$ | $3.8\pm0.4$ |
| 3 | Photofrin | 48 | $4.0\pm0.6$ | $5.1\pm0.4$ |
| 4 | HpD | 24 | NA | $4.6\pm0.1$ |
| 5 | 5-ALA[a] | 6 | 2.8 | >2.5 |

[a] Only one data point available. NA, not available.

**Table IV** Photodynamic threshold values for coagulative necrosis in normal brain and VX2 tumour: group average ± standard deviation.

| | | | $T_c$ $\times 10^{18}$ (photons $cm^{-3}$) | |
|---|---|---|---|---|
| Group | Photosensitiser | Treatment time (h) | VX2 tumour | Normal brain |
| 1 | Photofrin | 4-6 | $4.2\pm18$ | $2.8\pm1.5$ |
| 2 | Photofrin | 24 | $24\pm7$ | $2.2\pm1.1$ |
| 3 | Photofrin | 48 | $26\pm7$ | $1.2\pm0.4$ |
| 4 | HpD | 24 | NA | $2.5\pm1.6$ |
| 5 | 5-ALA[a] | 6 | 1.2 | 0.04–0.35[b] |

[a] Only one data point available. [b] Grey matter only. NA, not available. See text for definitions of the ranges with 5-ALA.

limit was calculated assuming $r_c = 6$ mm, which is the distance from the fibre tip to the dura. Studies to confirm these threshold values are in progress using surface illumination of the grey matter, which will allow accurate determination of the true depth of necrosis.

Using neuronal damage rather than necrosis as the biological end point results in lower threshold values: $(1.9\pm1.2,\ 1.8\pm0.8 \text{ and } 1.1\pm0.5)\times10^{18}$ photons $cm^{-3}$ at 4–6, 24 and 48 h, respectively, for Photofrin; $(2.1\pm1.2)\times10^{18}$ photons $cm^{-3}$ at 24 h for HpD.

For simplicity, the effect of photosensitiser photobleaching during light irradiation has not been taken into account in calculating the threshold values above. Extension of equation (1) to include photobleaching has been discussed previously (Wilson, 1992). An estimate of the effect can be obtained by considering the fraction of photosensitiser bleached at the radius of necrosis. For example, for Photofrin, the fluence at the radius of necrosis is approximately 10 J $cm^{-2}$ in both tumour and normal tissue (Figure 3), so that, for a photobleaching rate of 0.036 $cm^2$ $J^{-1}$ (Potter *et al.*, 1987), the fractional reduction is approximately 15%. Thus, in ignoring the photobleaching effect, the threshold values would be overestimated by approximately this amount.

### Discussion and conclusions

The use of the VX2 tumour in rabbits is costly and labour intensive, so that only a limited number of animals could be studied. While, for example, rat models have the potential to generate statistically significant data using a large number of animals (Chen *et al.*, 1992*a,b*; Dereski *et al.*, 1991), the rabbit model was chosen here since it provides a large enough cranial cavity to grow tumours of a suitable size and to generate measurable lesions by interstitial light delivery without acute morbidity. Additionally, the low-density to high-density lipoprotein ratio in New Zealand White rabbits is similar to that in humans, thus presenting a better model of photosensitiser pharmacokinetics (Chapman, 1986; Barclay, 1972). The choice of the rabbit model restricts the intracranial tumour type that can be used, and there is no glioma model available as there is for the rat (Kaye *et al.*, 1985; Dereski *et al.*, 1995*b*). Nevertheless, the uptake ratio of photosensitiser in tumour and BAT *vs* surrounding normal brain is similar to those reported for some rat glioma models: e.g. the tumour-to-normal ratio of Photofrin concentration at 48 h post injection was approximately 14 in a 9L glioma grown in Fischer rats (Dereski *et al.*, 1995*b*), the same as for VX2 in the rabbit brain. The PDT 'curability' of the intracranial VX2 is likely to be different from that of glioma. However, this and the small number of animals used, does not affect the major conclusions of the present study with respect to the PDT threshold and the qualitative comparison between tumour and normal tissue sensitivities. The known immunogenicity of the VX2 tumour in rabbits (Zagzag *et al.*, 1988) should also not alter the threshold values significantly, since the PDT-induced necrosis was measured at a short time interval post treatment.

In these experiments, the doses of photosensitiser used in the PDT-treated animals were high compared with clinical doses, as reviewed by Kostron *et al.* (1990), although similar to other preclinical studies (Dereski *et al.*, 1995*a,b*; Kaye and Morstyn, 1987). This kept the tissue concentration the same as for the uptake measurements, for which high doses gave improved accuracy. Thus, the tissue concentrations were directly applicable in calculating the threshold values, avoiding the need to assume linearity between administered dose and tissue concentration. Also, since the intent was to create sizeable zones of necrosis in order to measure the radii and fluences accurately, the higher concentration kept the treatment time to a minimum for fluence rates which did not cause tissue heating.

*Photofrin PDT threshold*

The Photofrin and HpD uptake measurements confirm earlier findings (Wilson *et al.*, 1988; Hill *et al.*, 1990; Ying *et al.*, 1992) of a very low volume uptake in normal brain. [Note that no steroids were administered before light activation, so that modification of the BBB, which could alter porphyrin uptake (Origitano *et al.*, 1993), need not be considered.] It is generally believed that the intact BBB reduces diffusion of Photofrin or HpD from the vasculature to the extravascular space. The highest concentrations of Photofrin in normal brain were found at short time intervals (4–6 h), possibly due to circulating drug (Peng *et al.*, 1991). The tissue concentration decreased at longer times, although about 50% of the peak concentration still remained at 48 h. In a mouse model the half-time for Photofrin clearance in plasma was reported to be approximately 5 h (Peng *et al.*, 1991), so that the contribution of circulating drug at 48 h can be neglected, and the concentration must be attributed to tissue-associated photosensitiser. Very slow clearance of Photofrin and HpD from normal human brain has also been reported in other studies (Boisvert *et al.*, 1985; Kaye *et al.*, 1985).

The time course of Photofrin concentration in the tumour was similar to that in normal brain, but with the peak uptake being about 12 times higher, attributable to breakdown of the BBB. Ying *et al.* (1992) noted in a rat glioma model that the uptake of photosensitiser into malignant tissue increases with progressive degradation of the BBB. As most human brain tumours are currently treated at a late stage, it can be assumed that the BBB is also disrupted, and similarly high uptake can be expected (Origitano *et al.*, 1993). Some investigators (Pottier and Kennedy, 1990; Woodburn *et al.*, 1992) have pointed out that, in poorly vascularised tumours, including human glioma (Kayama *et al.*, 1991), the tissue is more acidic, possibly increasing the uptake or retention of photosensitisers such as HpD and Photofrin.

Despite the very low volume uptake of Photofrin and HpD in normal brain, significant necrosis was produced at all times, resulting in a very low photodynamic threshold value compared with other normal tissues (Patterson *et al.*, 1990) (see Table V). The most likely explanation is that the intact BBB results in a very high concentration of photosensitiser in the capillary endothelium, and necrosis results from microvascular damage (Reed *et al.*, 1987). Our finding of subepithelial accumulations of fibrin and proteinaceous material adjacent to the PDT lesions suggest a subnecrotic effect of PDT on the vasculature.

The decrease of the photosensitiser concentration in normal brain and tumour at longer time intervals is not reflected in a decrease of the radii of necrosis. Thus, the threshold values decrease with time. This can only be explained by either a redistribution of photosensitiser to more sensitive target sites or selective removal of photosensitiser from less sensitive sites. Berenbaum *et al.* (1986) reported high sensitivity of normal rat brain to Photofrin PDT up to 84 days after injection and M Dereski (personal communication) found no change in the radius of Photofrin PTD-induced necrosis in normal rat brain several weeks after photosensitiser administration. Chen *et al.* (1992*a*,*b*) derived very low photodynamic threshold with Photofrin for transdural illumination of the grey matter in rats, suggesting different PDT sensitivity for white *vs* grey matter.

The relatively high Photofrin PDT threshold value in the well-vascularised intracranial VX2 tumour is similar to that in the poorly vascularised and poorly differentiated Dunning prostate tumour grown subcutaneously (M Olivo, unpublished results), and may be due to rate-limiting perfusion of oxygen in the intracranial tumour as the result of competitive oxygen use in adjacent brain tissue. This has been suggested by Lindsay *et al.* (1991), who observed reduced PDT response of malignant glioma in rats grown intracranially compared with extracranially. Comparative studies of the PDT threshold in the VX2 tumour under these conditions are in progress. Kayama *et al.* (1991) found that the intratumoral $pO_2$ was four times lower than in the brain cortex and up to eight times lower than in arterial blood, measured during surgery in humans. The arterial $paO_2$ measured in our experiments during PDT was close to four times lower than reported in the human study. Hence, very poorly oxygenated regions could be present in the VX2 tumour despite a good vascular supply, resulting in reduced sensitivity to photodynamic therapy (Foster *et al.*, 1991).

*Photofrin PDT* vs *HpD PDT threshold*

Kaye and Morstyn (1987) have reported smaller depths of necrosis of normal rat brain *vs* a glioma tumour model, treated using HpD under similar conditions, suggesting a higher threshold for necrosis in normal brain when using HpD compared with Photofrin. However, we observed no significant differences in the radius of necrosis in normal rabbit brain treated with either HpD or Photofrin. Parallel studies in a 9L glioma rat model (Q Chen and M Dereski, private communication) have found also no difference in the response of normal brain tissue to HpD and Photofrin. In the use of PDT for human patients, there appears to be a greater degree of normal brain response with Photofrin (Muller and Wilson, 1993) than with HpD (Kaye and Hill, 1993), as observed clinically. With Photofrin, elevated intracranial pressure, approximately double those of a resection-only control group, requiring high doses of steroids, have been reported (Muller and Wilson, 1993), suggesting significant cerebral oedema. This has not been reported to be a problem with HpD. Conversely, in neither case does there appear to be neurological deficit as result of the treatment, which is consistent with our finding that Photofrin and HpD have similar PDT effects on normal brain.

**Table V** Summary of photodynamic threshold values for comparison with Table IV

| *Tissue* | *Photosensitiser* | *Time interval (h)* | T $\times 10^{18}$ *(photons cm$^{-3}$)* | *Reference* |
|---|---|---|---|---|
| Liver (rat) | AISPc | 24 | 38 | Patterson *et al.* (1990) |
| | Photofrin | 24 | 3.5 | Farrell, (1991) |
| Human tumour (Head and neck) | Photofrin | 24 | 1.5[a] | Derived from Wenig *et al.* (1992) |
| Human tumour (various) | Photofrin | <72 | 0.86[a] | Derived from Potter, (1989) |
| Dunning prostate Tumour (rat flank) | AISPc | 24 | 20 | M Olivo (unpublished results) |
| Rat brain | Photofrin | 24 | 1.5[b,c] | Ying *et al.* (1994) |
| Rat brain | Photofrin | 48 | <1[c,d] | Chen *et al.* (1992*a*) and Dereski *et al.* (1991) |

[a] Applying assumed values for photosensitiser uptake and light penetration. [b] Assuming a photosensitiser concentration of $0.5\,\mu g\,g^{-1}$ tissue for $12.5\,mg\,kg^{-1}$ injected dose. [c] Necrosis limited mostly to grey matter. [d] Assuming a photosensitiser concentration of $0.2\,\mu g\,g^{-1}$ for $4\,mg\,kg^{-1}$ injected dose (based on the same specific uptake as Dereski *et al.*, (1991). AISPc, Aluminium chlorosulphonated phthalocyanine (tetrasulphonated).

*5-ALA PDT threshold*

*In vivo* synthesis of the photosensitiser PpIX from administration of 5-ALA is part of the haem synthesis pathway. Verma *et al.* (1993) showed that haem synthesis in normal rat brain is mainly restricted to the grey matter and olfactory bulb, possibly with the highest activity in the cortex. While Percy and Shanley (1977) showed that 5-ALA uptake in human cerebrospinal fluid is low for moderate blood serum concentrations (approximately 1 mg $l^{-1}$) during acute porphyria, McGillion *et al.* (1974) showed that 5-ALA can cross the BBB and accumulate in the cerebrospinal fluid of rats at elevated plasma concentration (20 mg $l^{-1}$). In this study, administering 5-ALA at 100 mg per kg bw i.v., we found a higher concentration of PpIX in tumour and a lower tumour-to-normal brain ratio than reported by Fukuda *et al.* (1992) after injecting liposomally encapsulated 5-ALA at 200 mg per kg bw i.v. into mice. This suggests that the low molecular weight 'free' 5-ALA can cross the intact BBB and is taken up by both normal and tumour tissues more efficiently than encapsulated 5-ALA. As white matter does not synthesise PpIX in high amounts, PDT treatment of deep-seated tumours in the white matter could be possible with a high therapeutic selectivity. This is also suggested by the lower tumour-to-normal threshold ratios for 5-ALA compared with the other photosensitisers. The extremely low threshold for regions of normal brain active in haem synthesis, including the grey matter, would require very careful application of PDT irradiation to spare these regions.

In conclusion, the photodynamic threshold model appears to be applicable to quantify the extent of PDT-induced tissue necrosis in intracranial tissues with the photosensitiser and irradiation parameters used here. The study has confirmed the high intrinsic PDT-sensitivity of normal brain. This is of concern when applying PDT as clinical treatment for human brain tumours. In order to achieve a good therapeutic effect with a wide margin of safety, the tumour-to-normal photodynamic threshold ratio should ideally be much less than unity. Since this is not the case, selective tumour destruction can only be achieved by having a high tumour-to-normal uptake ratio of photosensitiser and by accurate light targeting. Increase in the therapeutic ratio using alternative photosensitisers and/or modulating the PDT effect in normal brain is a worthwhile goal. Assuming that damage to normal brain is caused by vascular stasis, triggered by damage to the endothelium, protection might be achieved by clearance of the photosensitiser from the BBB before light treatment or bleaching of the photosensitiser at the onset of light treatment (Patterson and Wilson, 1994) to prevent endothelial cell damage, or suppression of clotting factor release after the endothelial cell layer is damaged (Ben Hur *et al.*, 1988), or by administration of thromboxane inhibitors to prevent vascular stasis (Fingar *et al.*, 1993). Detailed subcellular photosensitiser uptake studies, for example using confocal fluorescence microscopy (M Olivo, unpublished results), can play an important role in evaluating the possible efficacy of some of these interventions.

The observation of neuronal damage beyond the zone of coagulative damage in normal brain with Photofrin or HpD may also reduce the therapeutic efficacy of brain tumour PDT. The extent of neuron damage beyond coagulative necrosis becomes smaller for longer time delays between photosensitiser and light administration, favouring longer time delays (Muller and Wilson, 1993).

For Photofrin the maximum normal-to-tumour threshold ratio was obtained at 24 h. Of the five study groups, 5-ALA had the highest normal-to-tumour threshold ratio in white matter, and could be a very efficient photosensitiser for tumours seeded in this location. However, care will have to be taken to protect haem-synthesising structures in the brain from light irradiation, considering also that photoproducts are created when PpIX is light activated (Charlesworth and Truscott, 1993) and these could have different photoeffectiveness.

The large variation observed during light treatment in the local fluence rate and, hence, in the tissue optical properties emphasises the need for continuous fluence-rate monitoring during clinical PDT. The multifibre detector technique demonstrated might be well suited for deep-seated malignancies when non-invasive monitoring (Farrell *et al.*, 1992*b*; Farrell and Patterson, 1992) is not applicable.

Finally, the possible relevance of the present work to clinical PDT for treatment of malignant brain tumours should be considered. The low occurrence of reported major neurological deficits with Photofrin or HpD PDT in patients, (Muller and Wilson, 1993; Kaye and Hill, 1993) might be interpreted as challenging the present conclusion that normal brain has an intrinsically higher sensitivity to porphyrin-based PDT than brain tumour. However, it should be noted that the fraction of normal brain exposed during PDT in patients has generally been much less than in this rabbit model, even allowing for the fact that the clinical studies have been carried out using 630 nm light, where the penetration in tissue is much greater than at 514 nm. Thus, either there could be damage to normal human brain that is subclinical, or normal human brain does not have high sensitivity compared with brain tumour, or the high sensitivity in the normal rabbit brain is (partially) the result of the large fractional volume exposed or the relatively high photosensitiser doses used. Further studies to examine the last point are in progress. In the absence of evidence to the contrary, it also seems reasonable to proceed with further studies using the present model to examine possible alternative photosensitisers that may have significantly less effect on normal brain tissue and strategies to enhance the PDT sensitivity of intracranial tumour relative to surrounding normal brain. This is likely to be increasingly important as PDT is used to eradicate minimal residual tumour after surgical resection or small tumour foci without prior resection, when tumour selectivity will be critical.

**Acknowledgements**

This work was funded by the National Cancer Institute of Canada. One of the authors (LL) also acknowledges support through the National Institutes of Health (US) grant no. P01-CA43892. We wish to thank L Badousie for providing the VX2 tumour cell line, J Konieczny, J Wang and K Delaney for their assistance in the *in vivo* experiments, F Soares for preparation of histological sections, and T Farrell and P Muller for inspiring discussions. Photofrin was kindly supplied by Quadra Logic Technologies, Vancouver, BC, Canada, and HpD by A Kaye and J Hill, Royal Melbourne Hospital, Australia. All experiments were approved and carried out according to the guidelines of the ethics committee for animal care at McMaster University. L Lilge is currently with the Ontario Laser and Lightwave Research Center, Toronto, Ontario.

**Appendix**

The fluence at the boundary of necrosis can be calculated indirectly by applying a mathematical model of the light distribution in optically turbid media (Driver *et al.*, 1991; Arnfield *et al.*, 1992; Farrell and Patterson, 1992) based on average optical properties of the target tissue. In the present study, in addition to direct determination of the fluence at the radius of necrosis, the local fluence-rate measurements, $\Phi(r)$, at the three different radial distances, $r$, were used to derive the optical interaction coefficients of the target tissues, using a modified solution of the diffusion equation (Grosjean, 1956). This solution of the diffusion equation has been confirmed by Monte Carlo calculations close to the source and is equally accurate for scattering- or absorption-dominated tissues (Arnfield *et al.*, 1992). The reduced (transport) scattering coefficient, $\mu_s'$, and absorption coefficient, $\mu_a$, were derived as free parameters in a gridsearch $\chi$-square fit to the measured $\Phi(r)$ values and the mean delivered power before and after treatment. A geometrical correction for anisotropic delivery by the cut-end optical fibre was incorporated in the mathematical model (Lilge & Wilson, 1993). The effective

**Figure A1** Reduced scattering coefficient (○) and absorption coefficient (■) of tissues as a function of treatment time, derived from the local interstitial fluence-rate measurements shown in Figure 2. (**a**) Normal brain, Photofrin at 48 h. (**b**) Tumour, no photosensitiser. (**c**) Tumour, Photofrin at 4 h.

attenuation coefficient, $\mu_{eff}$, which determines the exponential component of the fluence profile, was calculated from the absorption and scattering coefficients, using diffusion theory (Patterson *et al*., 1991).

The resulting optical properties of normal rabbit brain and VX2 tumour are given in Figure A1 for each data set shown in Figure 2.

**Table AI** Optical properties of normal rabbit brain and VX2 tumour at 514 nm, pre-PDT group average ± standard deviation.

| | n | $\mu_a$ ($mm^{-1}$) | $\mu_s'$ ($mm^{-1}$) | *Indirect*[a] $\mu_{eff}$ ($mm^{-1}$) | *Direct*[b] $\mu_{eff}$ ($mm^{-1}$) |
|---|---|---|---|---|---|
| VX2 tumour | 12 | 0.092 ± 0.03 | 2.0 ± 1.1 | 0.7 ± 0.2 | 0.7 ± 0.1 |
| Normal brain | 12 | 0.11 ± 0.06 | 2.2 ± 1.3 | 0.9 ± 0.2 | 0.8 ± 0.3 |

[a] From $\mu_a$ and $\mu_s'$ values, determined from fitting the local fluence-rate to diffusion theory.
[b] Determined from the slope of $\ln[r\Phi(r)]$ at onset of PDT.

Variations in the local fluence-rate, as seen in Figure 2, are here reflected in these optical properties changing during the course of the treatment, Note that the sudden discontinuity seen in Figure 2 is not reflected in the derived optical properties values. The average values of $\mu_a$, $\mu_s'$ and $\mu_{eff}$ during light treatment are shown in Table AI. The effective attenuation coefficients alternatively derived directly from the semilogarithmic plots of $r$ H($r$) *vs* $r$, as shown in Figure 3, are also included in Table AI. The difference between the directly and indirectly determined values was generally less than 15%, in support of the mathematical model used to derive the absorption and scattering coefficients. In some animals the linear regression to calculate $\mu_{eff}$ directly and the fitting routine to derive $\mu_a$ and $\mu_s'$ showed significant errors, possibly due to individual detector fibres being close to strongly light-absorbing structures (e.g. blood vessels or blood clot). The precision of these interstitial fluence-rate measurements could be increased either by increasing the number of optical fibre detectors (Lilge and Wilson, 1993) or by a different geometrical arrangement of these around the source fibre.

The average optical properties and penetration depth ($1/\mu_{eff}$) of the 514 nm light in normal brain are similar to published values (Svaasand and Ellingsen, 1983; Chen *et al*., 1992*b*). Literature data for brain tumour are only available for 630 nm light, for which the penetration depth is substantially greater, 2.9 ± 1.5 mm (Muller and Wilson, 1986), than at 514 nm, due mainly to reduced haemoglobin absorption.

Monitoring of the fluence-rate during light treatment was found to be essential, as the fluence-rate distribution in brain tissue could alter unpredictably in the course of treatment, as seen in Figure 2 at particular times and locations. This type of sudden change in fluence rate was seen sporadically in a number of animals during treatment. It is not likely to be a technical artifact, such as movement of the fibre tip or changes in the fibre-detector coupling, since the change was usually transient and reversible. It is also not the result of a sudden loss or gain in delivered light, since this would be seen by all detectors equally. The most probable cause is local tissue disruption in the region of the individual detector fibre tip, such as an altered blood content in a nearby vessel or some bleeding around the detector, although the latter would not be rapidly reversible. The haemoglobin extinction coefficient is very high at 514 nm as used here, so that relatively small changes in local blood content could give marked fluence changes. We have also seen these effects in other tissues, such as the canine prostate (Q Chen, unpublished results).

Considering the slower, systematic trends in optical properties during treatment, reduced optical absorption due to photosensitiser photobleaching (Wilson *et al*., 1986) can be neglected in normal brain owing to the minimal contribution of the photosensitiser to the overall absorption coefficient (estimated at 0.0038 $cm^{-1}$ *vs* 0.11 $cm^{-1}$). However, it has to be considered for Photofrin and HpD in tumour tissue, as up to 15% of the overall absorption can be due to the photosensitiser. Changes in the scattering coefficient could be caused, for example, by oedema or fluid (e.g. cerebrospinal fluid) leaking into the target volume, or by incidental thermal damage to the tissue (King and Rudolf, 1993; Jaywant *et al*., 1993), although the latter is unlikely for the temperature changes observed. Direct monitoring of the fluence rate would enable compensation in clinical treatment time for such changes in the tissue optical properties due to the treatment itself.

### References

ALBRITTON EC. (1953). *Standard Values in Blood*. Saunders: Philadelphia.

ARNFIELD MR, MATHEW RP, TULIP J AND MCPHEE MS. (1992). Analysis of tissue optical coefficients using an approximate equation valid for comparable absorption and scattering. *Phys. Med. Biol*., **37**, 1219–1230.

BARCLAY M. (1972). Lipoprotein class distribution in normal and diseased states. In *Blood Lipids and Lipoproteins: Quantitation, Composition, and Metabolism*. Nelson GJ (ed.) pp 585–626.Wiley: New York.

BEN HUR E, HELDMAN E, CRANE SW AND ROSENTHAL I. (1988). Release of clotting factors from photosensitized endothelial cells, a possible trigger for blood vessel occlusion by photodynamic therapy. *FEBS Lett.*, **236**, 105–108.

BERENBAUM MCR, BONNETT R AND SCOURIDES PA. (1982). In vivo biological activity of the components of haematoporphyrin derivative. *Br. J. Cancer*, **45**, 571–581.

BERENBAUM MC, HALL GW AND HOYES AD. (1986). Cerebral photosensitation by hematoporphyrin derivative. Evidence for an endothelial site of action. *Br. J. Cancer*, **53**, 81–89.

BOGGAN JE, WALTER R, EDWARDS MSB, BORICHM JK, DAVIS RL, KOONCE M AND BERNS MW. (1984). Distribution of hematoporphyrin derivative in the rat 9L gliosarcoma brain tumor analyzed by digital video fluorescence microscopy. *J. Neurosurgery*, **61**, 113–119.

BOISVERT DPJ, MCKEAN JDS, TULIP J, CUMMINS J AND CHENG MK. (1985). Penetration of hematoporphyrin derivative into rat brain and intracerebral 9L glioma tissue. *J. Neuro-Oncol.*, **3**, 113–118.

BREM SS, ZAGZAG D, TSANACLIS AMC, GATELY S, ELKOUBY MP AND BRIEN SE. (1990). Inhibition of angiogenesis and tumour growth in the brain. *Am. J. Pathology*, **137**, 1121–1142.

CARSON BS, ANDERSON JH, GROSSMAN SA, HILTON J, WHITE CL, COLVIN OM, CLARK AW, GROCHOW LB, KAHN A AND MURRAY KJ. (1982). Improved rabbit brain tumour model amenable to diagnostic radiographic procedures. *Neurosurgery*, **11**, 603–608.

CHAPMAN MJ. (1986). Comparative analysis of mammalian plasma lipoprotein. *Methods Enzymol.*, **56**, 70–143.

CHARLESWORTH P AND TRUSCOTT TG. (1993). The use of 5-aminolevulinic acid (ALA) in photodynamic therapy (PDT). *Photochem. Photobiol.*, **57**, 99–100.

CHEN Q, WILSON BC, DERESKI MO, PATTERSON MS, CHOPP M AND HETZEL FW. (1992*a*). The effect of light beam size on fluence distribution and depth of necrosis in superficially applied photodynamic therapy of normal rat brain. *Photochem Photobiol.*, **56**, 379–384.

CHEN Q, CHOPP M, DERESKI MO, WILSON BC, PATTERSON MS, SCHREIBER A AND HETZEL FW. (1992*b*). The effect of light fluence rate in photodynamic therapy of normal rat brain. *Radiat. Res.*, **132**, 120–123.

CHENG MK, MCKEAN J, BOISVERT D, TULIP J AND MIELKE BW. (1984). Effects of photoradiation therapy on normal rat brain. *Neurosurgery*, **15**, 808–810.

DERESKI MO, CHOPP M, GARCIA JH AND HETZEL FW. (1991). Depth measurements and histopathological characterization of photodynamic therapy generated normal brain necrosis as a function of incident optical energy dose. *Photochem. Photobiol.*, **54**, 109–112.

DERESKI MO, MADIGAN L AND CHOPP M. (1995*a*). The effect of hypothermia and hyperthermia on PDT of normal brain. *Neurosurgery*, **36**, 58–64.

DERESKI MO, MADIGAN L AND CHOPP M. (1995*b*). 9L gliosarcoma sensitivity to PDT. *Neurosurgery* (in press).

DRIVER I, LOWDEL CP AND ASH DV. (1991). In vivo measurement of the optical interaction coefficients of human tumours at 630 nm. *Phys. Med. Biol.*, **36**, 805–813.

FARRELL TJ AND PATTERSON MS. (1992). A diffusion theory model of spatially resolved, steady-state diffuse reflectance for the noninvasive determination of tissue optical properties. *Med. Phys.*, **19**, 879–888.

FARRELL TJ, WILSON BC, PATTERSON MC AND CHOW R. (1991). The dependence of photodynamic threshold dose on treatment parameters in normal rat liver in vivo. *Proc. Soc. Photo-Opt. Instr. Eng.*, **1566**, 217–223.

FARRELL TJ, PATTERSON MS AND WILSON BC. (1992*a*). Investigation of the dependence of tissue necrosis on irradiation wavelength and time post injection using a photodynamic threshold dose model. In *Photodynamic Therapy*, Spinelli P, Dal Fante M, Marchesni R (eds) pp 830–843. Elsevier: New York.

FARRELL TJ, WILSON BC AND PATTERSON MS. (1992*b*). The use of a neural network to determine tissue optical properties from spatially resolved diffuse reflectance measurements. *Phys. Med. Biol.*, **37**, 2281–2286.

FINGAR VH, SIEGEL KA, WIEMAN TF AND WEBER-DOAK K. (1993). The effects of thromboxane inhibitors on the microvascular and tumor response to photodynamic therapy. *Photochem. Photobiol.*, **58**, 393–399.

FOSTER TH, MURANT RS, BRYANT RG, KNOX RS, GIBSON SL AND HILF R. (1991). Oxygen consumption and diffusion effects in photodynamic therapy. *Radiat. Res.*, **126**, 296–303.

FUKUDA H, PAREDES S AND BATTLE AM. (1992). Tumour-localizing properties of porphyrins. In vivo studies using free and liposome encapsulated aminolevulinic acid. *Comparative Biochem. Phys.*, **102**, 433–436.

GROSJEAN CC. (1956). A high accuracy approximation for solving multiple scattering problems in infinite homogeneous media *Nuovo Climento*, **3**, 1262–1275.

GUNTER EW, TURNER WE AND HUFF DL. (1989). Investigation of protoporphyrin IX standard materials used in acid-extraction methods and a proposed correction for the millimolar absorptivity of protoporphyrin IX. *Clin. Chem.*, **35**, 1601–1608.

HEBEDA KM, WOLBERS JG, STERENBORG HJCM, KAMPHORST W, GEMERT MJC VAN AND ALPHEN HAM VAN. (1995). Fluorescence localization in tumour and normal brain after intratumoral injection of haematoporphyrin derivative into rat brain tumour. *J. Photochem. Photobiol.*, **B27**, 85–92.

HILL JS, KAYE AH, SAWYER WH, MORSTYN G, MEGISON PD AND STYLLI SS. (1990). Selective uptake of hematoporphyrin derivative into human cerebral glioma. *Neurosurgery*, **26**, 248–254.

JAYWANT S, WILSON B, PATTERSON M, LILGE L, FLOTTE T, WOOLSEY J AND MCCULLOCH C. (1993). Temperature dependent changes in the optical absorption and scattering spectra of tissues: correlation with ultrastructure. *Proc. Soc. Photo-Opt. Instr. Eng.*, **1882**, 218–229.

KAYAMA T, YOSHIMOTO T, FUJIMOTO S AND SAKURAI Y. (1991). Intratumoral oxygen pressure in malignant brain tumor. *J. Neurosurgery*, **74**, 55–59.

KAYE AH. (1989). Photoradiation therapy of brain tumours, laboratory and clinical studies. In *Photosensitizing Compounds: Their Chemistry, Biology and Clinical Use. Ciba Foundation Symposium*, **146**, 206–224.

KAYE AH AND HILL JS. (1993). Photodynamic therapy of brain tumours. *Ann. Acad. Med. Singapore*, **22**, 470–481.

KAYE AH AND MORSTYN G. (1987). Photoradiation therapy causes selective tumor kill in a rat glioma model. *Neurosurgery*, **20**, 408–415.

KAYE AH, MORSTYN G AND ASHCROFT RG. (1985). Uptake and retention of hematoporphyrin derivative in an in vivo/in vitro model of cerebral glioma. *Neurosurgery*, **17**, 883–889.

KENNEDY JC AND POTTIER RH. (1992). Endogenous protoporphyrin IX, a clinically useful photosensitizer for photodynamic therapy. *J. Photochem. Photobiol.*, **B14**, 275–292.

KING WE AND RUDOLPH DB. (1993). Temperature rise in tumour tissue during high dose rate photoradiation. *Math. Biosciences*, **114**, 135–148.

KOSTRON H, PLANGGER C, FRITSCH E AND MAIER H. (1990). Photodynamic treatment of malingnant brain tumors. *Wiener klinische Wochenzeitschrift*, **102**, 531–535.

LEACH MW, KHOSHYOMN S, BRINGUS J, AUTRY SA AND BOGGAN JE. (1993). Normal brain tissue response to photodynamic therapy using aluminium phthalocyanine tetrasulfonate in the rat. *Photochem. Photobiol.*, **57**, 842–845.

LILGE L AND WILSON BC. (1993). The accuracy of interstitial measurements of absolute light fluence rate in the determination of tissue optical properties. *Proc. Soc. Photo-Opt. Instr. Eng.*, **1882**, 291–304.

LILGE L, OLIVO M, SCHATZ S AND WILSON BC. (1993*a*). Determination of the photodynamic threshold for normal rabbit brain and for intracranially implanted VX2 tumors. *Proc. Soc. Photo-Opt. Instr. Eng.*, **1882**, 60–72.

LILGE L, HAW T AND WILSON BC. (1993*b*). Miniature isotropic optical fibre probes for quantitative light dosimetry in tissue. *Phys. Med. Biol.*, **38**, 215–230.

LINDSAY EA, BERENBAUM MC, BONNETT R AND THOMAS DGT. (1991). Photodynamic therapy of a mouse glioma: Intracranial tumours are resistant while subcutaneous tumours are sensitive. *Br. J. Cancer*, **63**, 242–246.

MCGILLION FB, THOMPSON GG, MOORE MR AND GOLDBERG A. (1974). The passage of $\delta$-aminolaevulinic acid across the blood-brain barrier of the rat: Effect of ethanol. *Biochem. Pharm.*, **23**, 472–474.

MANG TS, DOUGHERTY TJ, POTTER WR, BOYLE DG, SOMMER S AND MOAN J. (1987). Photobleaching of porphyrins used in photodynamic therapy and implications for therapy. *J. Photochem. Photobiol.*, **A45**, 501–506.

MARCUS SL AND DUGAN MH. (1992). Global status of clinical photodynamic therapy: The registration process for a new therapy. *Lasers Surg. Med.*, **12**, 318–324.

MULLER PJ AND WILSON BC. (1986). An update on the penetration depth of 630 nm light in normal and malignant brain tissue *in vivo*. *Phys. Med. Biol.*, **31**, 1295–1297.
MULLER PJ AND WILSON BC. (1990). Photodynamic therapy of malignant brain tumours. *Can. J. Neurol. Sci.*, **17**, 193–198.
MULLER P AND WILSON B. (1991). Photodynamic therapy of brain tumours, post-operative 'field fractionation'. *J. Photochem. Photobiol.*, **B9**, 177–125.
MULLER PJ AND WILSON BC. (1993). Photodynamic therapy for brain tumours. In *Photodynamic Therapy of Malignancies*, McCaughan J (ed) pp 201–211. RC Landes: Austin, TX.
NOSKE DP, WOLBERS JG AND STERENBORG HJCM. (1991). Photodynamic therapy of malignant glioma. *Clin. Neurol. Neurosurg.*, **93**, 293–307.
ORIGITANO TC, KARESH SM, HENKIN RE, HALAMA JR AND REICHMAN OH. (1993). Photodynamic therapy for intracranial neoplasms: Investigations of photosensitizer uptake and distribution using indium 111 Photofrin II single photon emission computed tomography scans in humans with intracranial neoplasms. *Neurosurgery*, **32**, 357–364.
PATTERSON MS AND WILSON BC. (1994). A theoretical study of the influence of sensitizer photobleaching on depth of necrosis in photodynamic therapy. *Proc. Soc. Photo-Opt. Instr. Eng.*, **2133**, 208–219.
PATTERSON MS, WILSON BC AND GRAFF R. (1990). In vivo tests of the concept of photodynamic threshold dose in normal rat liver photosensitized by aluminium chlorosulphonated phthalocyanine. *Photochem. Photobiol.*, **51**, 343–349.
PATTERSON MS, WILSON BC AND WYMAN DR. (1991). The propagation of optical radiation in tissue I. Models of radiation transport and their application. *Lasers Med. Sci.*, **6**, 155–168.
PENG Q, MOAN J, KONGSHAUG M, EVENSEN JF, ANHOLT H AND RIMINGTON C. (1991). Sensitizer for photodynamic therapy of cancer: A comparison of the tissue distribution of Photofrin II and aluminum phthalocyanine tetrasulfonate in nude mice bearing a human malignant tumor. *Int. J. Cancer*, **48**, 258–264.
PERCY VA AND SHANLEY BC. (1977). Porphyrin precursors in blood, urine and cerebrospinal fluid in acute porphyria *S. Afr. Med. J.*, **52**, 219–222.
POTTER WR. (1989). PDT dosimetry and response. *Proc. Soc. Photo-Opt. Instr. Eng.*, **1065**, 88–99.
POTTER WR, MANG TS AND DOUGHERTY TJ. (1987). The theory of photodynamic therapy dosimetry: Consequences of photodestruction of sensitizer. *Photochem. Photobiol.*, **46**, 97–101.
POTTIER R AND KENNEDY JC. (1990). New trends in photobiology: The possible role of ionic species in selective biodistribution of photochemotherapeutic agents toward neoplastic tissue. *J. Photochem. Photobiol.*, **B8**, 1–16.
POWERS SK AND BROWN JT. (1986). Light dosimetry in brain tissue: An in vivo model applicable to photodynamic therapy. *Lasers Surg. Med.*, **6**, 318–322.
POWERS SK, CHUS SS, WALSTADT DL AND KWOCK L. (1991). Stereotactic intratumoral photodynamic therapy for recurrent malignant brain tumors. *Neurosurgery*, **29**, 688–696.
PROFIO AE AND DOIRON DR. (1981). Dosimetry considerations in phototherapy. *Med. Phys.*, **8**, 190–196.
REED MWRT, MILLER FN, WIEMAN TJ, TSENG MT AND PIETSCH CG. (1987). The effect of photodynamic therapy on the microcirculation. *J. Surg. Res.*, **45**, 452–459.
SANDEMAN DR, BRADFORD F, BUXTON P, BOWN SG AND THOMAS DGT. (1987). Selective necrosis of malignant gliomas in mice using photodynamic therapy. *Br. J. Cancer*, **55**, 647–649.
SVAASAND LO AND ELLINGSEN R. (1983). Optical properties of human brain. *Photochem. Photobiol.*, **38**, 293–299.
VERMA A, HIRSCH DJ, GLATT CE, RONNETT GV AND SNYDER SH. (1993). Carbon monoxide, a putative neural messenger. *Science*, **259**, 381–384.
VERNON DI, HOLYROD JHA, STIBBLING SM AND BROWN SB. (1995). The quantitative determination of photofrin and polyheamatoporphyrin in plasma: Pitfalls and inaccuracies. *J. Photochem. Photobiol.*, **B27**, 209–217.
WENIG BL, KIRTZMEN DM, GROSSWEINER LI, MAFEE MF, HARRIS DM, LOBRAICO RV, PRYCZ RA AND APPELBAUM EL. (1992). Photodynamic therapy in the treatment of squamous cell carcinoma of the head and neck. *Arch. Otolaryngol. Head Neck Surg.*, 116, 1267–1270.
WILSON BC. (1992). Optical and photobiological dosimetry for photodynamic therapy of solid tumour. In *Radiation Research: A Twentieth-Century Perspective. I.C.R.R.*, Vol. 2, Dewey WC, Edington M and Whitemore GF (eds) pp 647–679. Academic Press: New York.
WILSON BC, PATTERSON MS AND BURNS DM. (1986). Effect of photosensitiser concentration in tissue on the penetration depth of photoactivating light. *Lasers Med. Sci.*, **1**, 235–244.
WILSON BC, FIRNAU G, JEEVES WP, BROWN KL AND BURNS-MCCORMICK DM. (1988). Chromatographic analysis and tissue distribution of radiocopper-labeled haematoporphyrin derivatives. *Lasers Med. Sci.*, **3**, 71–80.
WOODBURN KW, STYLLI S, HILL JS, KAYE AH, REISS JA AND PHILLIPS DR. (1992). Evaluation of tumour and tissue distribution of porphyrins for use in photodynamic therapy. *Br. J. Cancer*, **65**, 321–328.
YING J, WALSTAD DL, BROWN JT AND POWERS SK. (1992). Relation between polyporphyrin distribution and blood brain barrier changes in the rat glioma model. *Lasers Surg. Med.*, **12**, 174–179.
YING JI, POWERS SK, BROWN JT, WALSTAD D AND MALINER L. (1994). Toxicity of photodynamic therapy with Photofrin in the normal rat brain. *Lasers Surg. Med.*, **14**, 219–228.
YOSHIDA Y, DERESKI MO, GARCIA JH, HETZEL FW AND CHOPP M. (1992*a*). Photoactivated Photofrin II, Astrocytic swelling precedes endothelial injury in rat brain. *J. Neuropathology*, **51**, 91–100.
YOSHIDA Y, DERESKI MO, GARCIA JH, HETZEL FW AND CHOPP M. (1992*b*). Neuronal injury after photoactivation of Photofrin II. *Am. J. Pathol.*, **141**, 989–997.
ZAGZAG D, BREM S AND ROBERT F. (1988). Neovascularization and tumor growth in the rabbit brain. *Am. J. Pathol.*, **131**, 361–377.


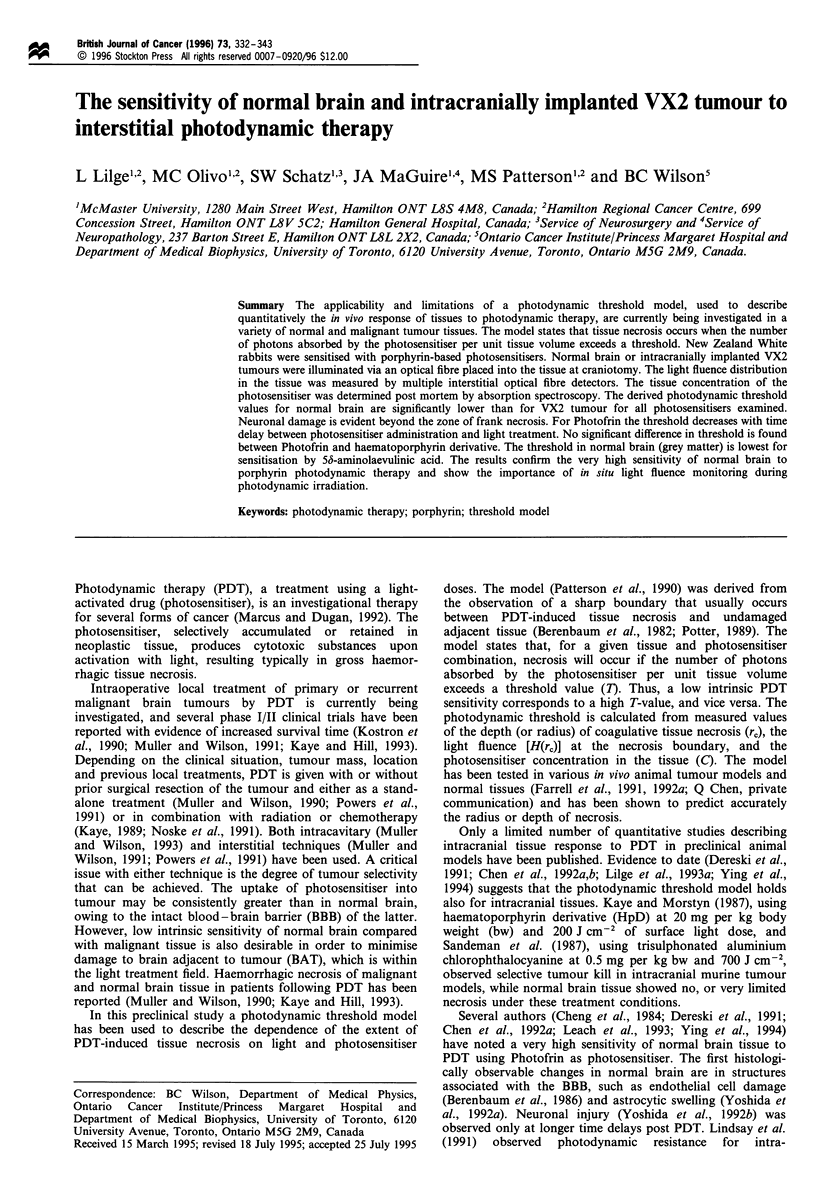

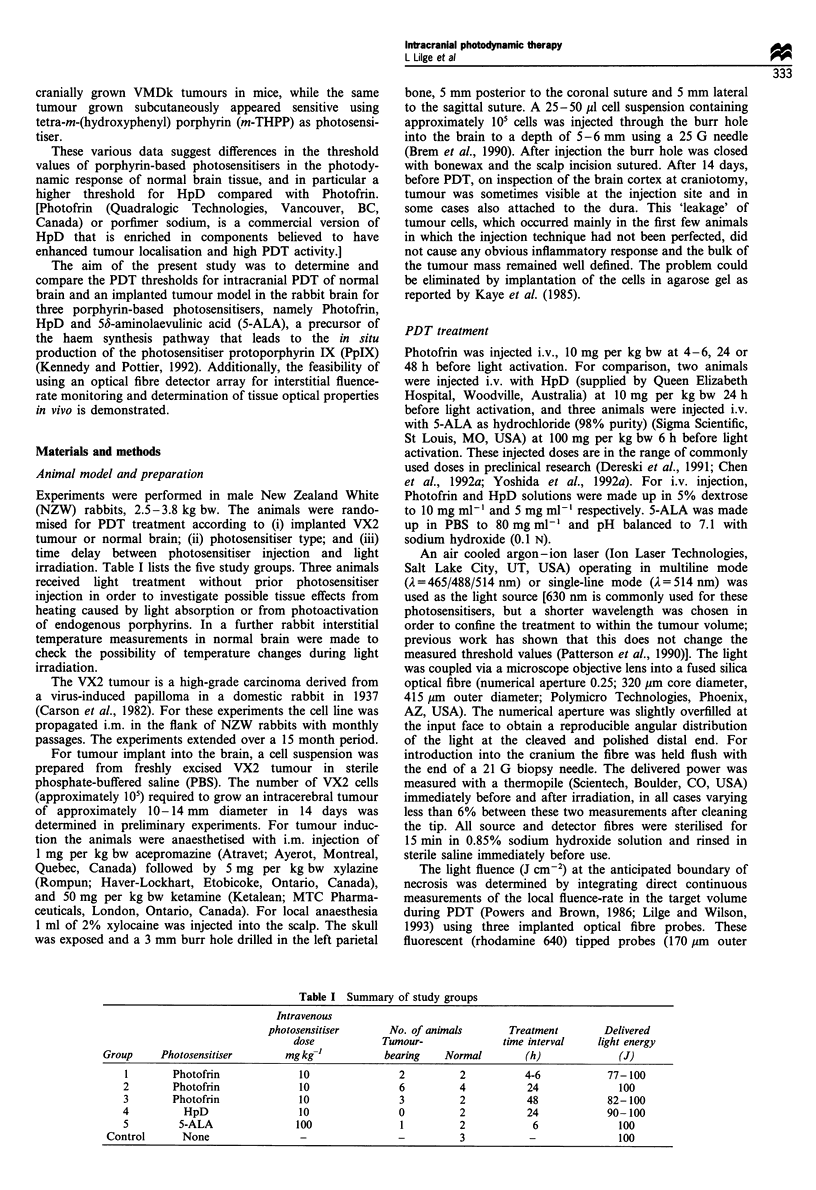

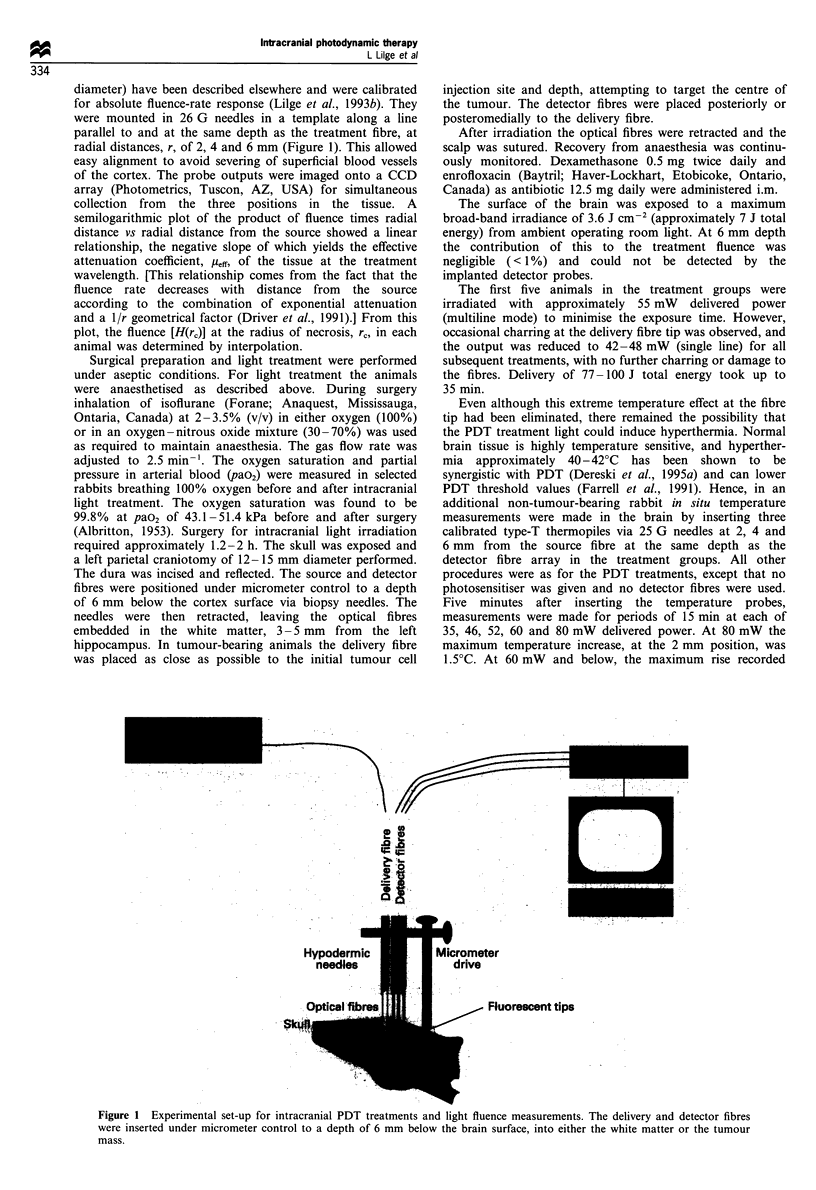

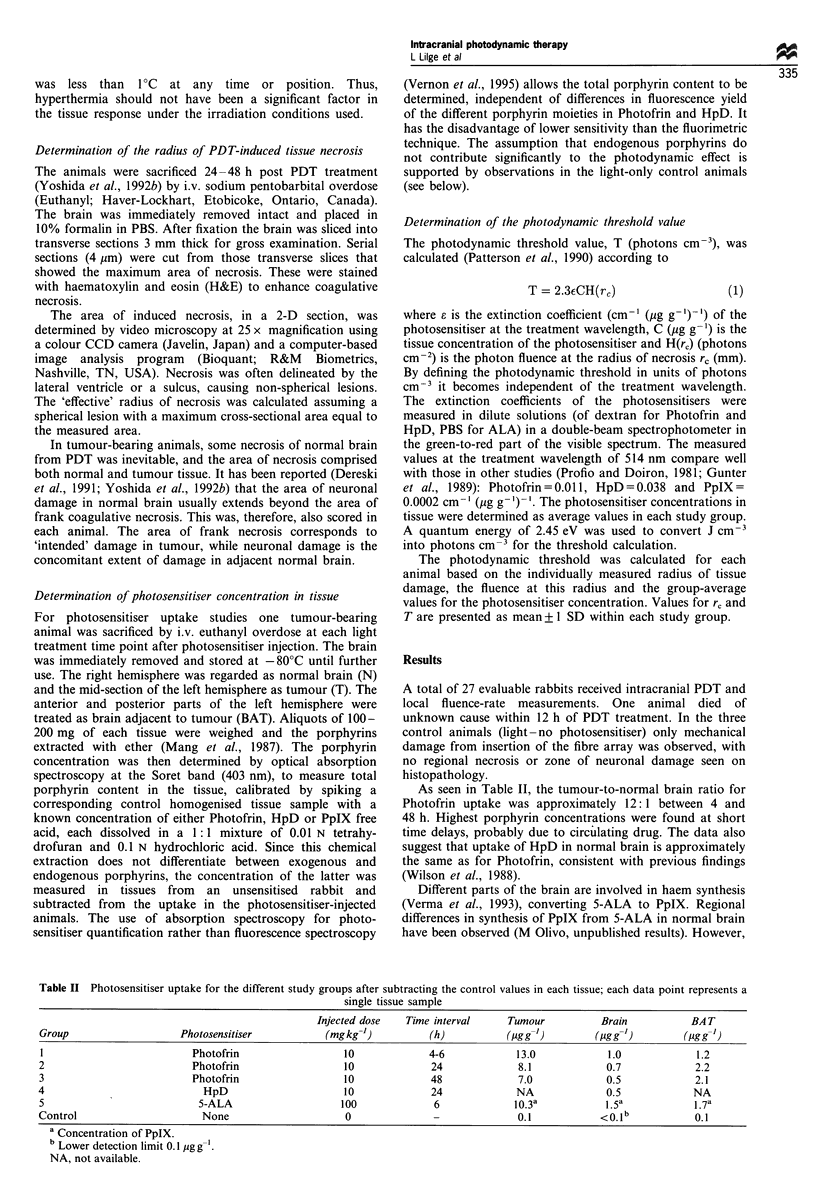

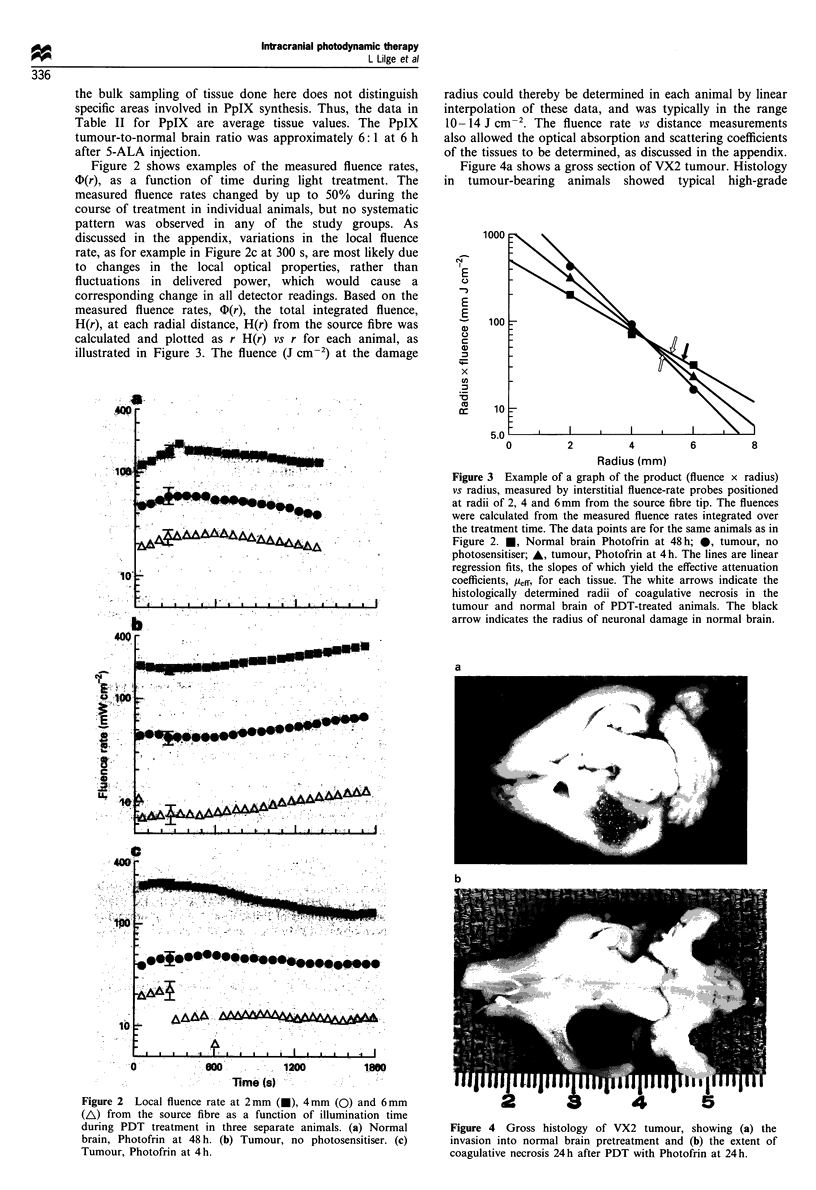

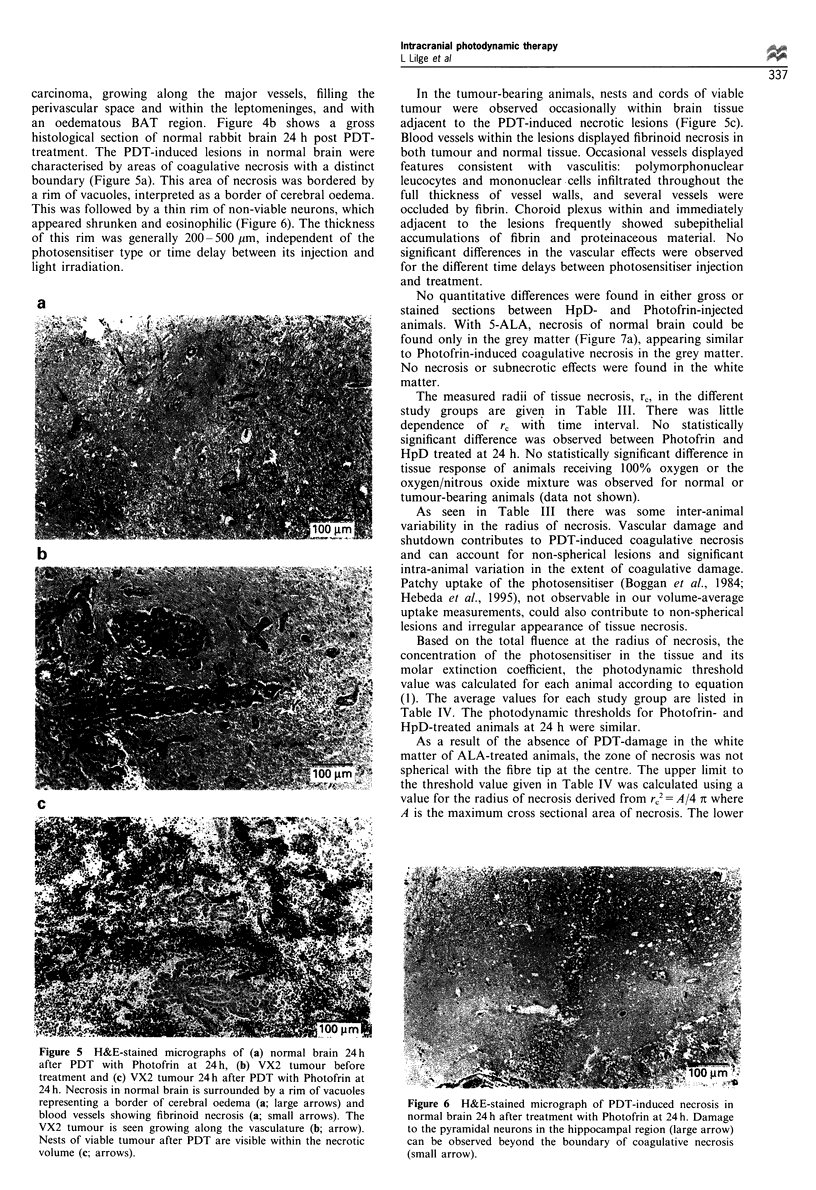

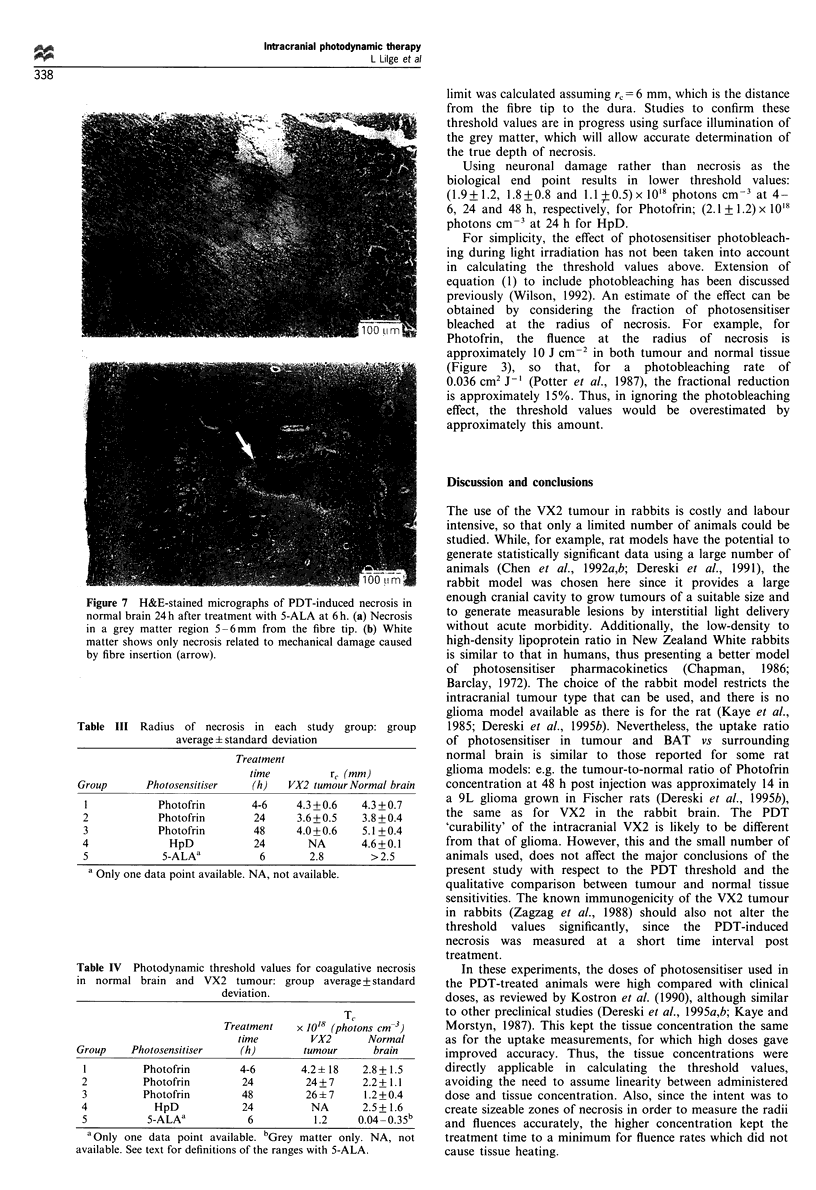

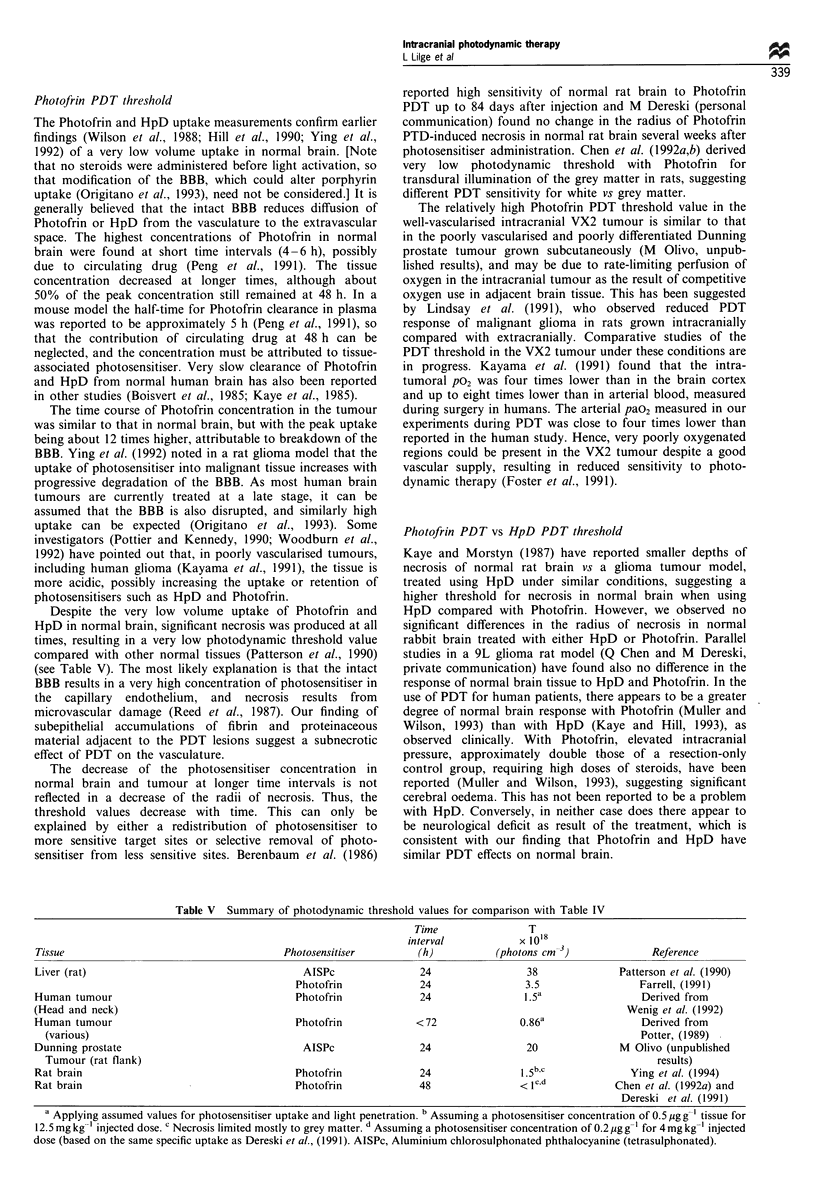

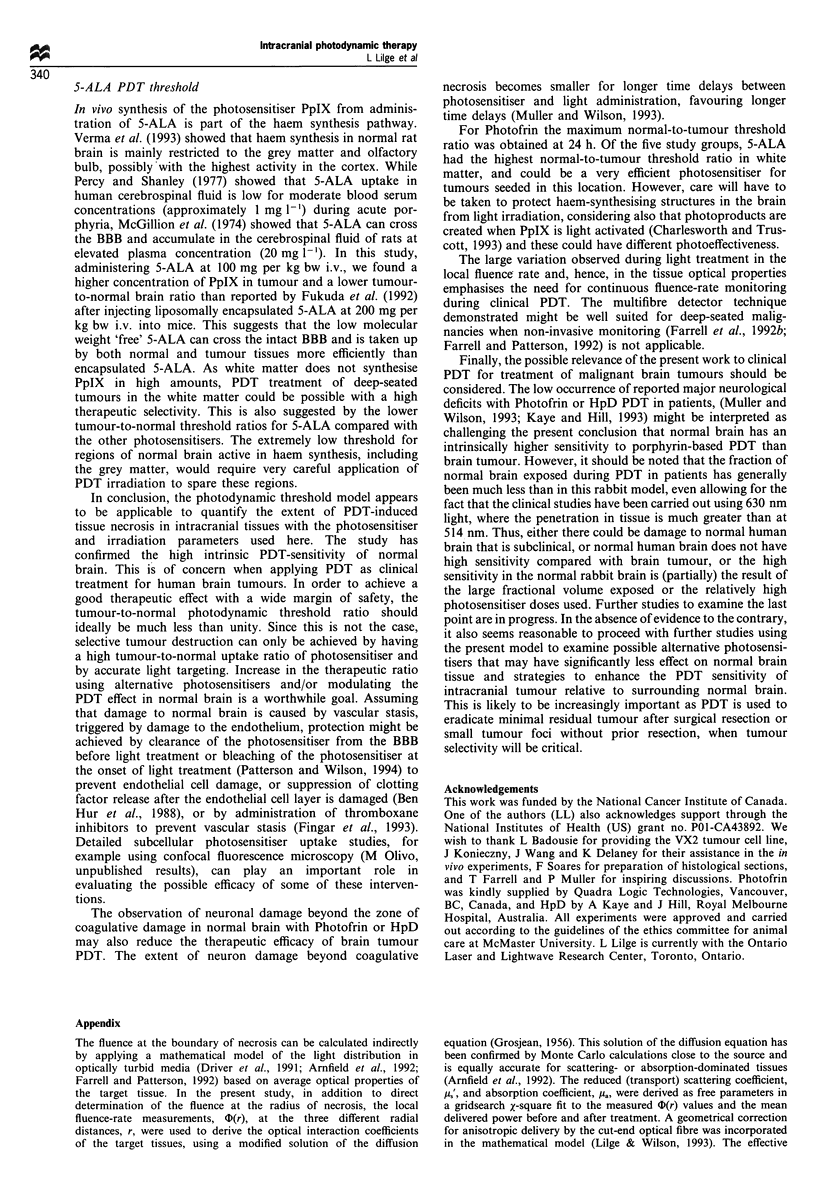

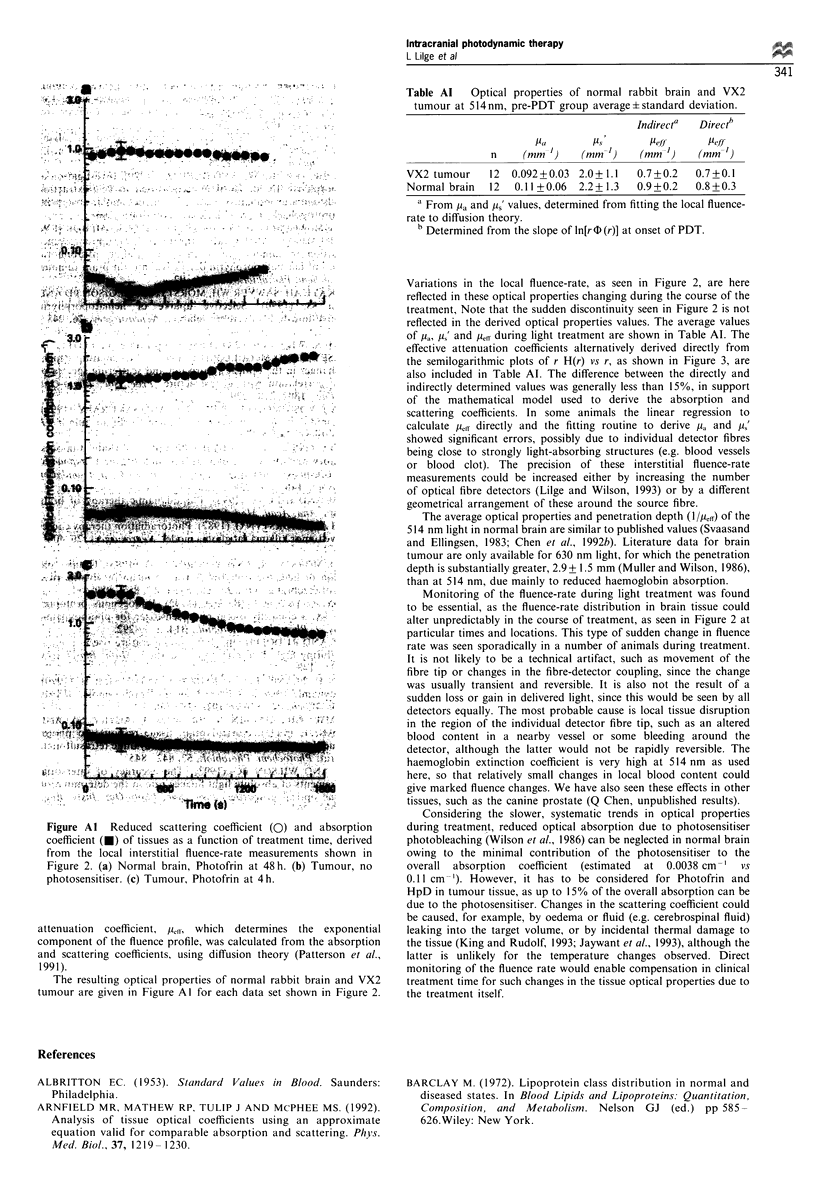

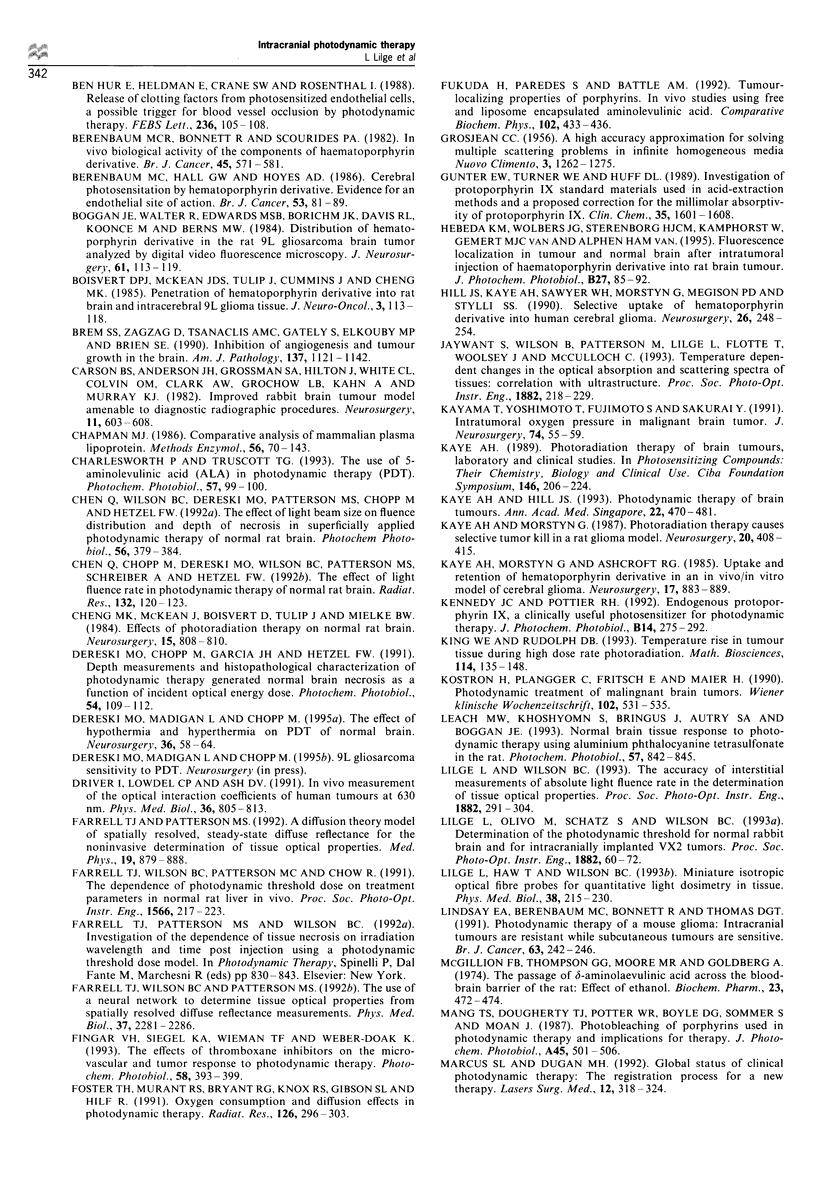

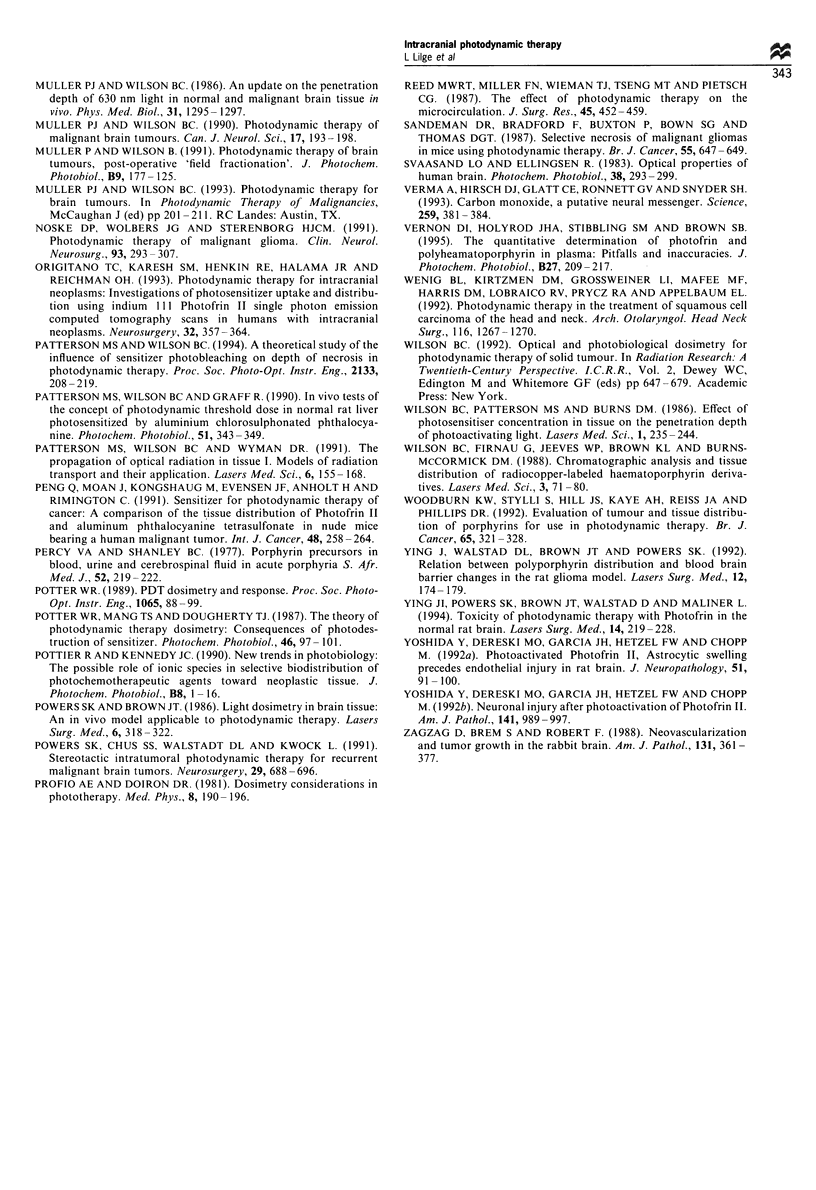


## References

[OCR_01365] Arnfield M. R., Mathew R. P., Tulip J., McPhee M. S. (1992). Analysis of tissue optical coefficients using an approximate equation valid for comparable absorption and scattering.. Phys Med Biol.

[OCR_01381] Ben-Hur E., Heldman E., Crane S. W., Rosenthal I. (1988). Release of clotting factors from photosensitized endothelial cells: a possible trigger for blood vessel occlusion by photodynamic therapy.. FEBS Lett.

[OCR_01389] Berenbaum M. C., Bonnett R., Scourides P. A. (1982). In vivo biological activity of the components of haematoporphyrin derivative.. Br J Cancer.

[OCR_01394] Berenbaum M. C., Hall G. W., Hoyes A. D. (1986). Cerebral photosensitisation by haematoporphyrin derivative. Evidence for an endothelial site of action.. Br J Cancer.

[OCR_01406] Boisvert D. P., McKean J. D., Tulip J., Cummins J., Cheng M. K. (1985). Penetration of hematoporphyrin derivative into rat brain and intracerebral 9L glioma tissue.. J Neurooncol.

[OCR_01410] Brem S. S., Zagzag D., Tsanaclis A. M., Gately S., Elkouby M. P., Brien S. E. (1990). Inhibition of angiogenesis and tumor growth in the brain. Suppression of endothelial cell turnover by penicillamine and the depletion of copper, an angiogenic cofactor.. Am J Pathol.

[OCR_01415] Carson B. S., Anderson J. H., Grossman S. A., Hilton J., White C. L., Colvin O. M., Clark A. W., Grochow L. B., Kahn A., Murray K. J. (1982). Improved rabbit brain tumor model amenable to diagnostic radiographic procedures.. Neurosurgery.

[OCR_01422] Chapman M. J. (1986). Comparative analysis of mammalian plasma lipoproteins.. Methods Enzymol.

[OCR_01440] Chen Q., Chopp M., Dereski M. O., Wilson B. C., Patterson M. S., Schreiber A., Hetzel F. W. (1992). The effect of light fluence rate in photodynamic therapy of normal rat brain.. Radiat Res.

[OCR_01433] Chen Q., Wilson B. C., Dereski M. O., Patterson M. S., Chopp M., Hetzel F. W. (1992). Effects of light beam size on fluence distribution and depth of necrosis in superficially applied photodynamic therapy of normal rat brain.. Photochem Photobiol.

[OCR_01446] Cheng M. K., McKean J., Boisvert D., Tulip J., Mielke B. W. (1984). Effects of photoradiation therapy on normal rat brain.. Neurosurgery.

[OCR_01449] Dereski M. O., Chopp M., Garcia J. H., Hetzel F. W. (1991). Depth measurements and histopathological characterization of photodynamic therapy generated normal brain necrosis as a function of incident optical energy dose.. Photochem Photobiol.

[OCR_01467] Driver I., Lowdell C. P., Ash D. V. (1991). In vivo measurement of the optical interaction coefficients of human tumours at 630 nm.. Phys Med Biol.

[OCR_01472] Farrell T. J., Patterson M. S., Wilson B. (1992). A diffusion theory model of spatially resolved, steady-state diffuse reflectance for the noninvasive determination of tissue optical properties in vivo.. Med Phys.

[OCR_01491] Farrell T. J., Wilson B. C., Patterson M. S. (1992). The use of a neural network to determine tissue optical properties from spatially resolved diffuse reflectance measurements.. Phys Med Biol.

[OCR_01497] Fingar V. H., Siegel K. A., Wieman T. J., Doak K. W. (1993). The effects of thromboxane inhibitors on the microvascular and tumor response to photodynamic therapy.. Photochem Photobiol.

[OCR_01501] Foster T. H., Murant R. S., Bryant R. G., Knox R. S., Gibson S. L., Hilf R. (1991). Oxygen consumption and diffusion effects in photodynamic therapy.. Radiat Res.

[OCR_01508] Fukuda H., Paredes S., Batlle A. M. (1992). Tumour-localizing properties of porphyrins. In vivo studies using free and liposome encapsulated aminolevulinic acid.. Comp Biochem Physiol B.

[OCR_01519] Gunter E. W., Turner W. E., Huff D. L. (1989). Investigation of protoporphyrin IX standard materials used in acid-extraction methods, and a proposed correction for the millimolar absorptivity of protoporphyrin IX.. Clin Chem.

[OCR_01526] Hebeda K. M., Wolbers J. G., Sterenborg H. J., Kamphorst W., van Gemert M. J., van Alphen H. A. (1995). Fluorescence localization in tumour and normal brain after intratumoral injection of haematoporphyrin derivative into rat brain tumour.. J Photochem Photobiol B.

[OCR_01532] Hill J. S., Kaye A. H., Sawyer W. H., Morstyn G., Megison P. D., Stylli S. S. (1990). Selective uptake of hematoporphyrin derivative into human cerebral glioma.. Neurosurgery.

[OCR_01785] Ji Y., Powers S. K., Brown J. T., Walstad D., Maliner L. (1994). Toxicity of photodynamic therapy with photofrin in the normal rat brain.. Lasers Surg Med.

[OCR_01781] Ji Y., Walstad D. L., Brown J. T., Powers S. K. (1992). Relation between polyporphyrin distribution and blood brain barrier changes in the rat glioma model.. Lasers Surg Med.

[OCR_01545] Kayama T., Yoshimoto T., Fujimoto S., Sakurai Y. (1991). Intratumoral oxygen pressure in malignant brain tumor.. J Neurosurg.

[OCR_01554] Kaye A. H., Hill J. S. (1993). Photodynamic therapy of brain tumours.. Ann Acad Med Singapore.

[OCR_01565] Kaye A. H., Morstyn G., Ashcroft R. G. (1985). Uptake and retention of hematoporphyrin derivative in an in vivo/in vitro model of cerebral glioma.. Neurosurgery.

[OCR_01560] Kaye A. H., Morstyn G. (1987). Photoradiation therapy causing selective tumor kill in a rat glioma model.. Neurosurgery.

[OCR_01570] Kennedy J. C., Pottier R. H. (1992). Endogenous protoporphyrin IX, a clinically useful photosensitizer for photodynamic therapy.. J Photochem Photobiol B.

[OCR_01573] King W. E., Rudolph D. B. (1993). Temperature rise in tumor tissue during high-dose-rate photoradiation.. Math Biosci.

[OCR_01580] Kostron H., Plangger C., Fritsch E., Maier H. (1990). Photodynamic treatment of malignant brain tumors.. Wien Klin Wochenschr.

[OCR_01585] Leach M. W., Khoshyomn S., Bringus J., Autry S. A., Boggan J. E. (1993). Normal brain tissue response to photodynamic therapy using aluminum phthalocyanine tetrasulfonate in the rat.. Photochem Photobiol.

[OCR_01603] Lilge L., Haw T., Wilson B. C. (1993). Miniature isotropic optical fibre probes for quantitative light dosimetry in tissue.. Phys Med Biol.

[OCR_01608] Lindsay E. A., Berenbaum M. C., Bonnett R., Thomas D. G. (1991). Photodynamic therapy of a mouse glioma: intracranial tumours are resistant while subcutaneous tumours are sensitive.. Br J Cancer.

[OCR_01618] Mang T. S., Dougherty T. J., Potter W. R., Boyle D. G., Somer S., Moan J. (1987). Photobleaching of porphyrins used in photodynamic therapy and implications for therapy.. Photochem Photobiol.

[OCR_01626] Marcus S. L., Dugan M. H. (1992). Global status of clinical photodynamic therapy: the registration process for a new therapy.. Lasers Surg Med.

[OCR_01614] McGillion F. B., Thompson G. G., Moore M. R., Goldberg A. (1974). The passage of delta-aminolaevulinic acid across the blood-brain barrier of the rat: effect of ethanol.. Biochem Pharmacol.

[OCR_01634] Muller P. J., Wilson B. C. (1986). An update on the penetration depth of 630 nm light in normal and malignant human brain tissue in vivo.. Phys Med Biol.

[OCR_01639] Muller P. J., Wilson B. C. (1990). Photodynamic therapy of malignant brain tumours.. Can J Neurol Sci.

[OCR_01655] Noske D. P., Wolbers J. G., Sterenborg H. J. (1991). Photodynamic therapy of malignant glioma. A review of literature.. Clin Neurol Neurosurg.

[OCR_01658] Origitano T. C., Karesh S. M., Henkin R. E., Halama J. R., Reichman O. H. (1993). Photodynamic therapy for intracranial neoplasms: investigations of photosensitizer uptake and distribution using indium-111 Photofrin-II single photon emission computed tomography scans in humans with intracranial neoplasms.. Neurosurgery.

[OCR_01674] Patterson M. S., Wilson B. C., Graff R. (1990). In vivo tests of the concept of photodynamic threshold dose in normal rat liver photosensitized by aluminum chlorosulphonated phthalocyanine.. Photochem Photobiol.

[OCR_01683] Peng Q., Moan J., Kongshaug M., Evensen J. F., Anholt H., Rimington C. (1991). Sensitizer for photodynamic therapy of cancer: a comparison of the tissue distribution of Photofrin II and aluminum phthalocyanine tetrasulfonate in nude mice bearing a human malignant tumor.. Int J Cancer.

[OCR_01690] Percy V. A., Shanley B. C. (1977). Prophyrin precursors in blood, urine and cerebrospinal fluid in acute porphyria.. S Afr Med J.

[OCR_01699] Potter W. R., Mang T. S., Dougherty T. J. (1987). The theory of photodynamic therapy dosimetry: consequences of photo-destruction of sensitizer.. Photochem Photobiol.

[OCR_01704] Pottier R., Kennedy J. C. (1990). The possible role of ionic species in selective biodistribution of photochemotherapeutic agents toward neoplastic tissue.. J Photochem Photobiol B.

[OCR_01710] Powers S. K., Brown J. T. (1986). Light dosimetry in brain tissue: an in vivo model applicable to photodynamic therapy.. Lasers Surg Med.

[OCR_01715] Powers S. K., Cush S. S., Walstad D. L., Kwock L. (1991). Stereotactic intratumoral photodynamic therapy for recurrent malignant brain tumors.. Neurosurgery.

[OCR_01722] Profio A. E., Doiron D. R. (1981). Dosimetry considerations in phototherapy.. Med Phys.

[OCR_01726] Reed M. W., Miller F. N., Wieman T. J., Tseng M. T., Pietsch C. G. (1988). The effect of photodynamic therapy on the microcirculation.. J Surg Res.

[OCR_01732] Sandeman D. R., Bradford R., Buxton P., Bown S. G., Thomas D. G. (1987). Selective necrosis of malignant gliomas in mice using photodynamic therapy.. Br J Cancer.

[OCR_01735] Svaasand L. O., Ellingsen R. (1983). Optical properties of human brain.. Photochem Photobiol.

[OCR_01739] Verma A., Hirsch D. J., Glatt C. E., Ronnett G. V., Snyder S. H. (1993). Carbon monoxide: a putative neural messenger.. Science.

[OCR_01744] Vernon D. I., Holroyd J. A., Stribbling S. M., Brown S. B. (1995). The quantitative determination of Photofrin and Polyhaematoporphyrin in plasma: pitfalls and inaccuracies.. J Photochem Photobiol B.

[OCR_01748] Wenig B. L., Kurtzman D. M., Grossweiner L. I., Mafee M. F., Harris D. M., Lobraico R. V., Prycz R. A., Appelbaum E. L. (1990). Photodynamic therapy in the treatment of squamous cell carcinoma of the head and neck.. Arch Otolaryngol Head Neck Surg.

[OCR_01776] Woodburn K. W., Stylli S., Hill J. S., Kaye A. H., Reiss J. A., Phillips D. R. (1992). Evaluation of tumour and tissue distribution of porphyrins for use in photodynamic therapy.. Br J Cancer.

[OCR_01796] Yoshida Y., Dereski M. O., Garcia J. H., Hetzel F. W., Chopp M. (1992). Neuronal injury after photoactivation of photofrin II.. Am J Pathol.

[OCR_01790] Yoshida Y., Dereski M. O., Garcia J. H., Hetzel F. W., Chopp M. (1992). Photoactivated Photofrin II: astrocytic swelling precedes endothelial injury in rat brain.. J Neuropathol Exp Neurol.

[OCR_01801] Zagzag D., Brem S., Robert F. (1988). Neovascularization and tumor growth in the rabbit brain. A model for experimental studies of angiogenesis and the blood-brain barrier.. Am J Pathol.

