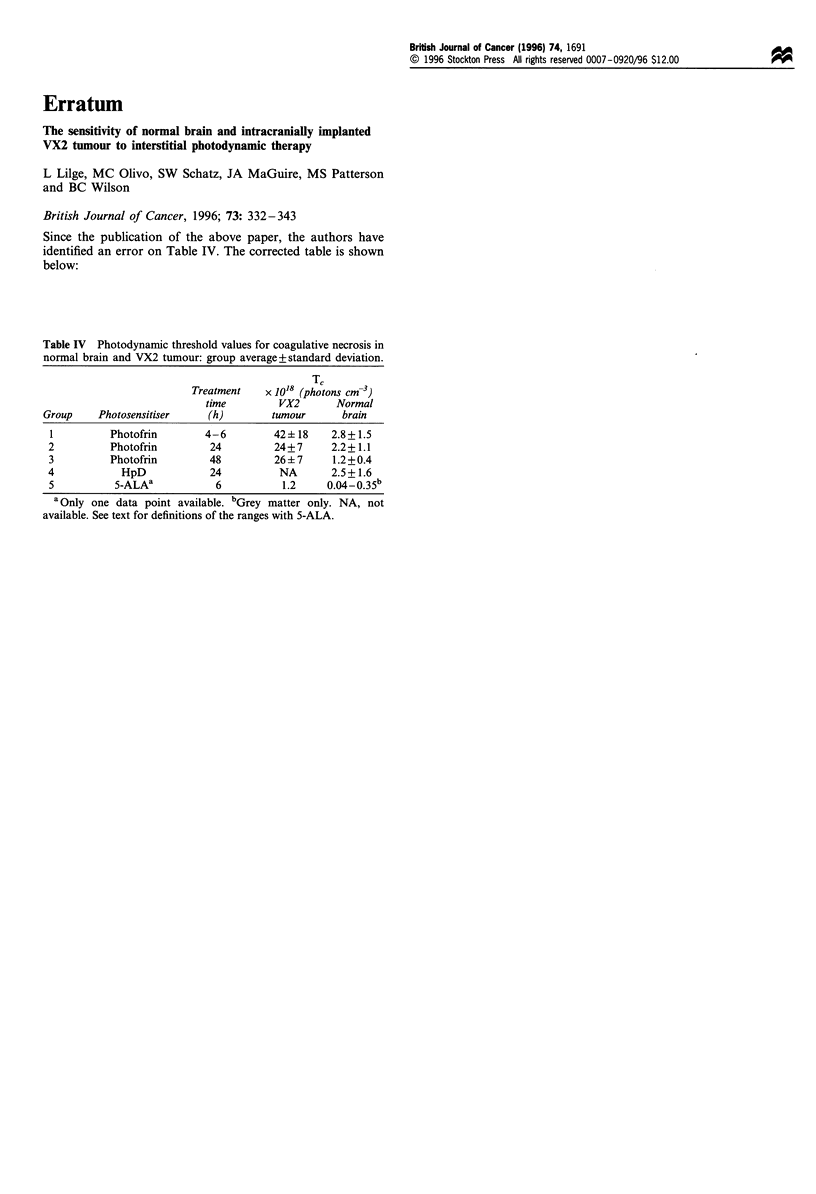# The sensitivity of normal brain and intracranially implanted VX2 tumour to interstitial photodynamic therapy

**Published:** 1996-11

**Authors:** 


**British Journal of Cancer (1996) 74, 1691**
© 1996 Stockton Press All rights reserved 0007-0920/96 $12.00

### Erratum

**The sensitivity of normal brain and intracranially implanted VX2 tumour to interstitial photodynamic therapy**

L Lilge, MC Olivo, SW Schatz, JA MaGuire, MS Patterson and BC Wilson

*British Journal of Cancer*, 1996; **73:** 332–343

Since the publication of the above paper, the authors have identified an error on Table IV. The corrected table is shown below:

**Table IV** Photodynamic threshold values for coagulative necrosis in normal brain and VX2 tumour: group average±standard deviation.

| | | | $T_c$ $\times 10^{18}$ (photons $cm^{-3}$) | |
|---|---|---|---|---|
| *Group* | *Photosensitiser* | *Treatment time (h)* | *VX2 tumour* | *Normal brain* |
| 1 | Photofrin | 4–6 | 42±18 | 2.8±1.5 |
| 2 | Photofrin | 24 | 24±7 | 2.2±1.1 |
| 3 | Photofrin | 48 | 26±7 | 1.2±0.4 |
| 4 | HpD | 24 | NA | 2.5±1.6 |
| 5 | 5-ALA[a] | 6 | 1.2 | 0.04–0.35[b] |

[a]Only one data point available. [b]Grey matter only. NA, not available. See text for definitions of the ranges with 5-ALA.